# Gene dysregulation analysis builds a mechanistic signature for prognosis and therapeutic benefit in colorectal cancer

**DOI:** 10.1093/jmcb/mjaa041

**Published:** 2020-07-27

**Authors:** Quanxue Li, Wentao Dai, Jixiang Liu, Qingqing Sang, Yi-Xue Li, Yuan-Yuan Li

**Affiliations:** 1 School of Biotechnology, East China University of Science and Technology, Shanghai 200237, China; 2 Shanghai Center for Bioinformation Technology, Shanghai 201203, China; 3 Department of Surgery, Shanghai Key Laboratory of Gastric Neoplasms, Shanghai Institute of Digestive Surgery, Ruijin Hospital, Shanghai Jiao Tong University School of Medicine, Shanghai 200025, China; 4 CAS Key Laboratory of Computational Biology, CAS-MPG Partner Institute for Computational Biology, Shanghai Institutes for Biological Sciences, Chinese Academy of Sciences, Shanghai 200031, China; 5 Shanghai Engineering Research Center of Pharmaceutical Translation and Shanghai Industrial Technology Institute, Shanghai 201203, China

**Keywords:** gene dysregulation analysis, mechanistic signature, cancer precision medicine, prognosis, chemotherapy benefit, colorectal cancer

## Abstract

The implementation of cancer precision medicine requires biomarkers or signatures for predicting prognosis and therapeutic benefits. Most of current efforts in this field are paying much more attention to predictive accuracy than to molecular mechanistic interpretability. Mechanism-driven strategy has recently emerged, aiming to build signatures with both predictive power and explanatory power. Driven by this strategy, we developed a robust gene dysregulation analysis framework with machine learning algorithms, which is capable of exploring gene dysregulations underlying carcinogenesis from high-dimensional data with cooperativity and synergy between regulators and several other transcriptional regulation rules taken into consideration. We then applied the framework to a colorectal cancer (CRC) cohort from The Cancer Genome Atlas. The identified CRC-related dysregulations significantly covered known carcinogenic processes and exhibited good prognostic effect. By choosing dysregulations with greedy strategy, we built a four-dysregulation (4-DysReg) signature, which has the capability of predicting prognosis and adjuvant chemotherapy benefit. 4-DysReg has the potential to explain carcinogenesis in terms of dysfunctional transcriptional regulation. These results demonstrate that our gene dysregulation analysis framework could be used to develop predictive signature with mechanistic interpretability for cancer precision medicine, and furthermore, elucidate the mechanisms of carcinogenesis.

## Introduction

Biomarkers or signatures for predicting prognosis and therapeutic benefits are an indispensable part for implementing cancer precision medicine (Walther et al., 2009; Vargas and Harris, 2016). The improvements of prognostic and therapeutic benefits with the aid of signatures have been reported in colorectal cancer (CRC), breast cancer, lung cancer, etc. (Chen et al., 2007; Khambata-Ford et al., 2007; Salazar et al., 2011; Sparano et al., 2019). Meta-analysis studies of clinical trials demonstrate that response rate seen with targeted agents under biomarker guidance has reached ∼30%, which is much higher than that of chemotherapies (Schwaederle et al., 2015). However, despite these promising outcomes, there remain urgent needs for improving the performance of clinical signatures.

The most widely adopted strategies for identifying predictive signatures heavily depend on expression analysis of individual genes involving identification of differentially expressed genes (DEGs) (Khambata-Ford et al., 2007; Salazar et al., 2011) and genes whose expression values are relevant to certain phenotype (Chen et al., 2007; Sparano et al., 2019). It is apparent that they ignore gene interconnection implicated in transcriptomic data, even though genes perform their functions in coordination, instead of in isolation (Barzel and Barabasi, 2013). Therefore, the currently available predictive signatures unavoidably tend to have limited mechanism explanatory power, which has become a common concern in precision medicine (Robinson et al., 2013). There is a general consensus among both clinicians and biologists about the need for signatures with mechanistic interpretability as well as high predictive accuracy for cancer precision medicine (Robinson et al., 2013; Topalian et al., 2016; Lu et al., 2019). It could also be expected that taking mechanistic interpretation into consideration would further enhance the predictive accuracy and robustness of signatures in clinical application (Robinson et al., 2013).

Attributed to the crucial roles of gene regulation in fundamental cell processes, to build signatures with mechanistic interpretability requires studying relevant genes in the context of regulatory networks, that is, identifying specific regulators and their regulatory relationships that are dysfunctional in a given disease state (Lee and Young, 2013). In the past >10 years, quite a few investigations have aimed to elucidate dysfunctional regulatory networks in disease, instead of solely focusing on DEGs (de la Fuente, 2010; Ideker and Krogan, 2012). Among them, differential coexpression analysis (DCA, also shortened as ‘DCEA’ in literatures), which were developed to identify differences in genes coexpression patterns between healthy and disease samples, was regarded as the first steps toward differential regulation analysis or gene dysregulation analysis (de la Fuente, 2010). Most of the existing DCA-based methodologies construct coexpression networks and identify the alteration of gene‒gene identities (i.e. network topology) or gene‒gene correlation (i.e. edge weight) (Ideker and Krogan, 2012; Li et al., 2016). Based on these strategies, differential modules or gene sets related to certain phenotypes could be identified to build signatures for predicting prognosis (Taylor et al., 2009) and drug response (Zickenrott et al., 2016). However, most signatures involve too many genes and include too much noise, which greatly weaken their mechanism explanatory power. In our early work, we applied our previously developed DCA-based method to cancer and generated a series of carcinogenesis-relevant biomarkers (Liu et al., 2010; Yu et al., 2011; Yang et al., 2013; Wu et al., 2016; Li et al., 2017a; Dai et al., 2018). Benefiting from the quantitative design of our DCA-based algorithms and candidate gene screening by transcriptional regulation relationships, the numbers of genes in our biomarkers were decreased to a practical level and the mechanistic interpretability of the identified biomarkers were also elevated (Wu et al., 2016; Li et al., 2017a).

Since correlation analysis cannot distinguish direct associations from indirect associations (Barzel and Barabasi, 2013), it has limited potential to directly provide clues into disease mechanisms. Aiming to identify the alterations of gene regulation relationships instead of expression correlations between various phenotypes, updated versions of differential regulation analysis have emerged as a follow-up effort toward elucidating disease-related dysfunctional regulatory networks or differential regulation relationships (Cao et al., 2015; Li et al., 2017b). Considering that transcriptional regulation requires sufficient cooperativity and synergy of multiple regulators (Lambert et al., 2018), however, it is still a huge challenge about how to robustly build a framework for dysregulation analysis based on high-dimensional transcriptome data if taking cooperativity and synergy of multiple regulators into consideration.

**Figure 1:**
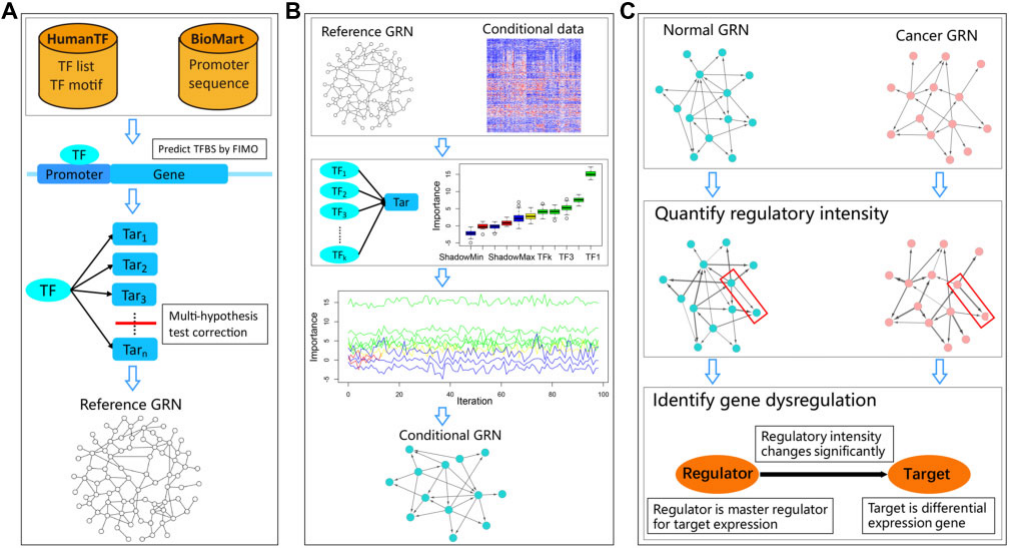
The framework of gene dysregulation analysis. (**A**) Construction of reference GRN by predicting the binding sites of TFs within promoter regions with TF motif data. (**B**) Construction of conditional GRNs with *Boruta* algorithm. (**C**) Identification of gene dysregulations by integrating three standards that regulatory intensity changes significantly between conditions, the target is a differential expression gene, and TF is a master regulator for target expression.

In this work, we first proposed a framework for gene dysregulation analysis by using machine learning algorithms that are able to consider the cooperativity and synergy between regulators and robustly cope with high-dimensional data (Figure 1). A reference gene regulation network (GRN) was constructed by predicting the potential binding site of transcription factors (TFs) among promoter regions (Figure 1A). Conditional GRNs were then highlighted with a random forest-based feature selection algorithm *Boruta* (Kursa and Rudnicki, 2010; Figure 1B), and each link’s regulatory intensity and its confidential interval were estimated with a de-biased least absolute shrinkage and selection operator (LASSO) method (Javanmard and Montanari, 2014; Figure 1C). Gene dysregulations were subsequently identified by integrating three properties including differential regulation, differential expression of target, and the consistency between differential regulation and differential expression (Figure 1C). We applied the framework to CRC, one of the most incident malignancies and the leading causes of cancer death around the world (Bray et al., 2018). The identified CRC-related dysregulations significantly covered well-known carcinogenic processes and exhibited good prognostic effect. Furthermore, a signature was constructed based on the dysregulations, which possessed not only predictive power for prognosis and adjuvant chemotherapy (ADJC) benefit but also mechanism explanatory power in terms of dysfunctional gene regulation. We provide our gene dysregulation analysis framework to the community and hope that this will help researchers generate mechanistic signatures with high predictive accuracy for cancer prognosis and treatment and gain insights into carcinogenesis.

## Results

### Identification of gene dysregulations

**Figure 2:**
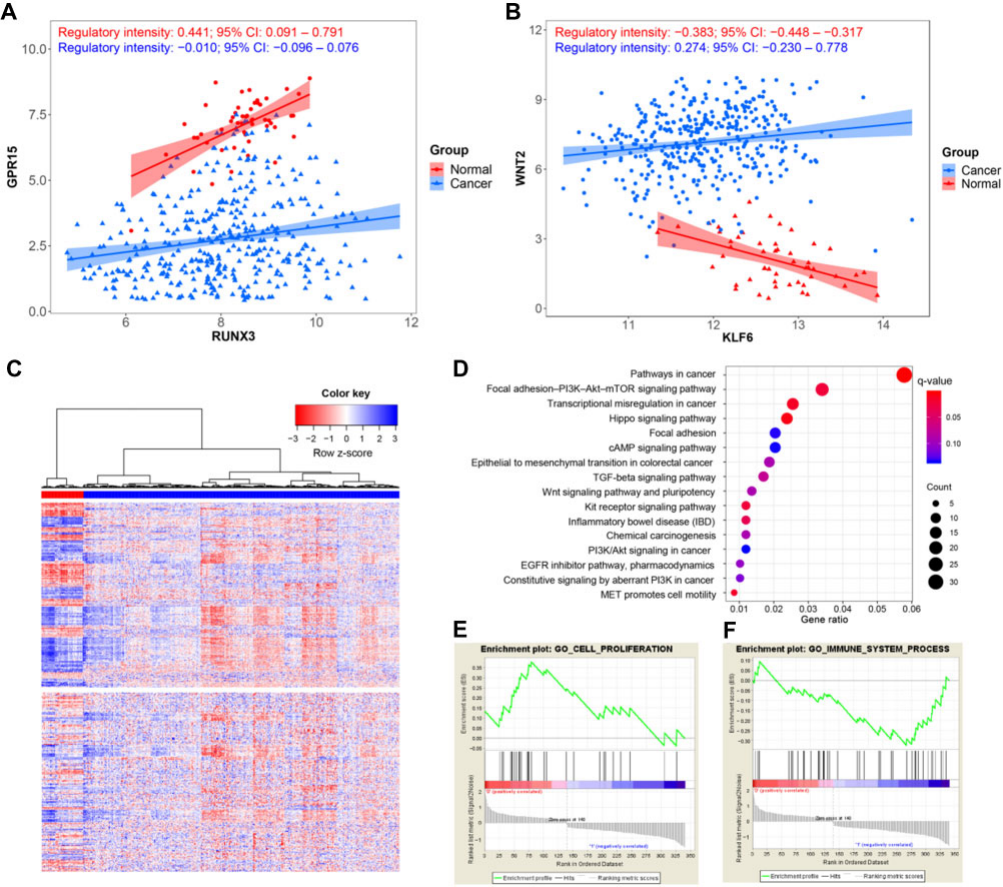
Summary of the identified gene dysregulations in CRC. (**A** and **B**) Examples of gene dysregulations. (**A**) RUNX3→GPR15. (**B**) KLF6→WNT2. X-axis denotes TF expression level and y-axis denotes target expression level. One point corresponds to one sample, with red representing normal and blue representing cancer. The regression lines and confidence interval shadows were calculated by single variable regression and used to visualize gene regulation differences between conditions. (**C**) Heatmap of gene expression of identified dysregulations. Upper, expression of targets; lower, expression of TFs. All the samples hold the same rank. Red represents normal samples, while blue represents cancer samples. (**D**) Cancer-related pathways in over-representation analysis. (**E** and **F**) The pathways with changed activity between normal and cancer exported by GSEA. (**E**) Cell proliferation. (**F**) Immune system process.

First of all, conditional GRNs, i.e. normal GRN and cancer GRN, were constructed by using *Boruta* algorithm (Kursa and Rudnicki, 2010) based on candidate TF‒target relationships and mRNA expression data of 32 paired samples from The Cancer Genome Atlas (TCGA) CRC dataset (Vivian et al., 2017). A total of 30186 and 15665 regulations were eventually kept in normal GRN and cancer GRN, respectively. The regulatory intensities and their 95% confidence intervals (CIs) of every link in conditional GRNs were quantified by de-biased LASSO (Javanmard and Montanari, 2014; see Supplementary File for validation of the quantifying method of regulatory intensity). Subsequently, 389 gene dysregulations were extracted according to three factors including differential regulation, differential expression of target, and the consistency between differential regulation and differential expression (Supplementary Table S1). Two examples of gene dysregulation, RUNX3→GPR15 and KLF6→WNT2, were illustrated in Figure 2. For RUNX3→GPR15 (Figure 2A), the regulatory intensity was reduced from normal to cancer with the intensity value being 0.441 (normal) and −0.010 (cancer), and the expression level of the target, GPR15, significantly decreased as shown. For KLF6→WNT2 (Figure 2B), the regulatory intensity was increased from normal (−0.383) to cancer (0.274), and accordingly, the expression level of the target, WNT2, was elevated. It was interesting that either the target set (341 genes) or the TF set (262 genes) involved in dysregulations was able to correctly classify tumor and normal samples with unsupervised hierarchical clustering method, suggesting that the 389 gene dysregulations were potentially relevant to carcinogenesis (Figure 2C;  Supplementary Figure S1).

To globally understand the functions of the 389 dysregulations, we implemented pathway over-representation analysis and obtained numerous cancer-related pathways enriched for the dysregulation genes (Figure 2D;  Supplementary Table S2). In order to explore the influences of dysregulation events on carcinogenesis, we extracted the targets’ expression data and checked the change of pathway activities with gene set enrichment analysis (GSEA) (Subramanian et al., 2005). It was shown that several biological processes including cell proliferation, cell cycle, pathways in cancer, chromosome organization, and vasculature development were more active in CRC samples (Figure 2E for cell proliferation; Supplementary Figure S2 for all), while some others including immune system process, cell death, and cell–cell adhesion were inhibited in cancer (Figure 2F for immune system process; Supplementary Figure S2 for all). These results support that the identified 389 dysregulations essentially highlight cancer-related crucial processes and thus could be taken as functional seeds for building explanatory signature.

### Prognostic effects of gene dysregulations

**Figure 3:**
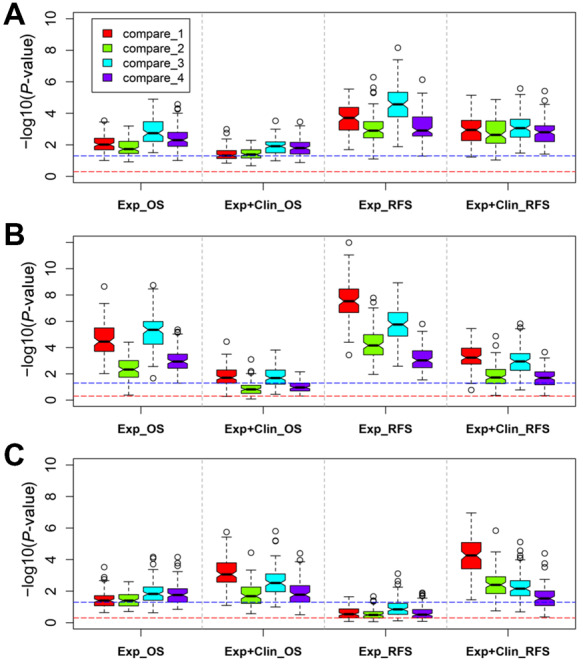
Prognostic effects of the identified gene dysregulations. (**A**) Results in TCGA CRC dataset. (**B**) Results in GSE39582 dataset. (**C**) Results in GSE17538 dataset. X-axis indicates four prognostic models. Exp+Clin_OS: OS cox model fitted with two genes’ expression data and clinical information; Exp_OS: OS cox model fitted with two genes’ expression data; Exp+Clin_RFS: RFS cox model fitted with two genes’ expression data and clinical information; Exp_RFS: RFS cox model fitted with two genes’ expression data. Y-axis indicates negative logarithm of *P*-value of Wilcox test when examining whether C-indexes of cox models fitted with dysregulations were significantly larger than controls. Each test was repeated 100 times. The blue dashed line indicates negative logarithm of 0.05. The red dashed line indicates negative logarithm of 0.5. compare_1, compare_2, compare_3, and compare_4 represent four types of control settings. compare_1: two genes were randomly selected from the gene list of preprocessed expression data; compare_2: one DEG and one non-DEG were randomly selected from the gene list of preprocessed expression data; compare_3: one regulation was randomly selected from the reference GRN; compare_4: one regulation whose target is DEG was randomly selected from the reference GRN.

We first checked prognostic effects of the 389 gene dysregulations for overall survival/recurrent-free survival (OS/RFS) on TCGA CRC dataset. Following the procedures in Materials and methods, we found that for all four types of cox models, C-indexes of 389 models fitted with the 389 dysregulations were significantly larger than those with 389 gene pairs randomly selected through four gene selection strategies (see Materials and methods for details), with the median *P*-value of 100 times of Wilcox tests <0.05 (Figure 3A). We then tested the prognostic effect of the 389 dysregulations in two independent datasets, GSE39582 and GSE17538. In GSE39582, similar trends were obtained excepting the OS cox model fitted with expression data and clinical information when the control setting involved DEGs (Figure 3B, comparison_2 and comparison_4 in Exp+Clin_OS). In GSE17538, similar trends were also observed apart from the RFS cox model fitted with expression data alone (Figure 3C, Exp_RFS). These results indicate that the 389 CRC-related dysregulations have prognostic capability as a whole.

### Construction of prognostic signature with dysregulations

Considering that our identified 389 dysregulations could cover known carcinogenic processes (Figure 2) and exhibit prognostic effect (Figure 3), we set out to construct a high-accuracy prognostic model based on these dysregulations, which was expected to provide both mechanism explanatory power and predictive power. First of all, among the 389 OS cox models fitted with expression data of 389 dysregulations adjuvant with clinical information from TCGA CRC dataset (Supplementary Table S3), the model for RUNX3→GPR15 dysregulation stood out with the largest C-index, 0.763. RUNX3, a member of the runt domain-containing family of TFs, has been found to be essential for diverse processes including proliferation, differentiation, cell lineage specification, apoptosis, and DNA repair (Ito et al., 2015; Bae et al., 2019). RUNX3 is taken as a tumor suppressor in CRC (Weisenberger et al., 2006; Soong et al., 2009). GPR15, an orphan G-protein-linked receptor, was reported to mediate T-cells localization to colon (Habtezion et al., 2016), and very recently proposed to represent a therapeutic target for CRC (Namkoong et al., 2019).

Given that plasma membrane proteins show great potential as drug targets and diagnostic objectives, we narrowed down the 389 dysregulations to 126 plasma membrane protein-relevant dysregulations to build a prognostic signature with explanatory capability (Supplementary Table S3). Starting from RUNX3→GPR15, a greedy strategy as described in Materials and methods was used to select other dysregulations to boost the signature’s performance. At last, a prognostic signature involving four dysregulations (4-DysReg), RUNX3→GPR15, RUNX3→P2RY8, SNAI3→TLR7, and ATOH1→SIGLEC1, was built.

**Figure 4:**
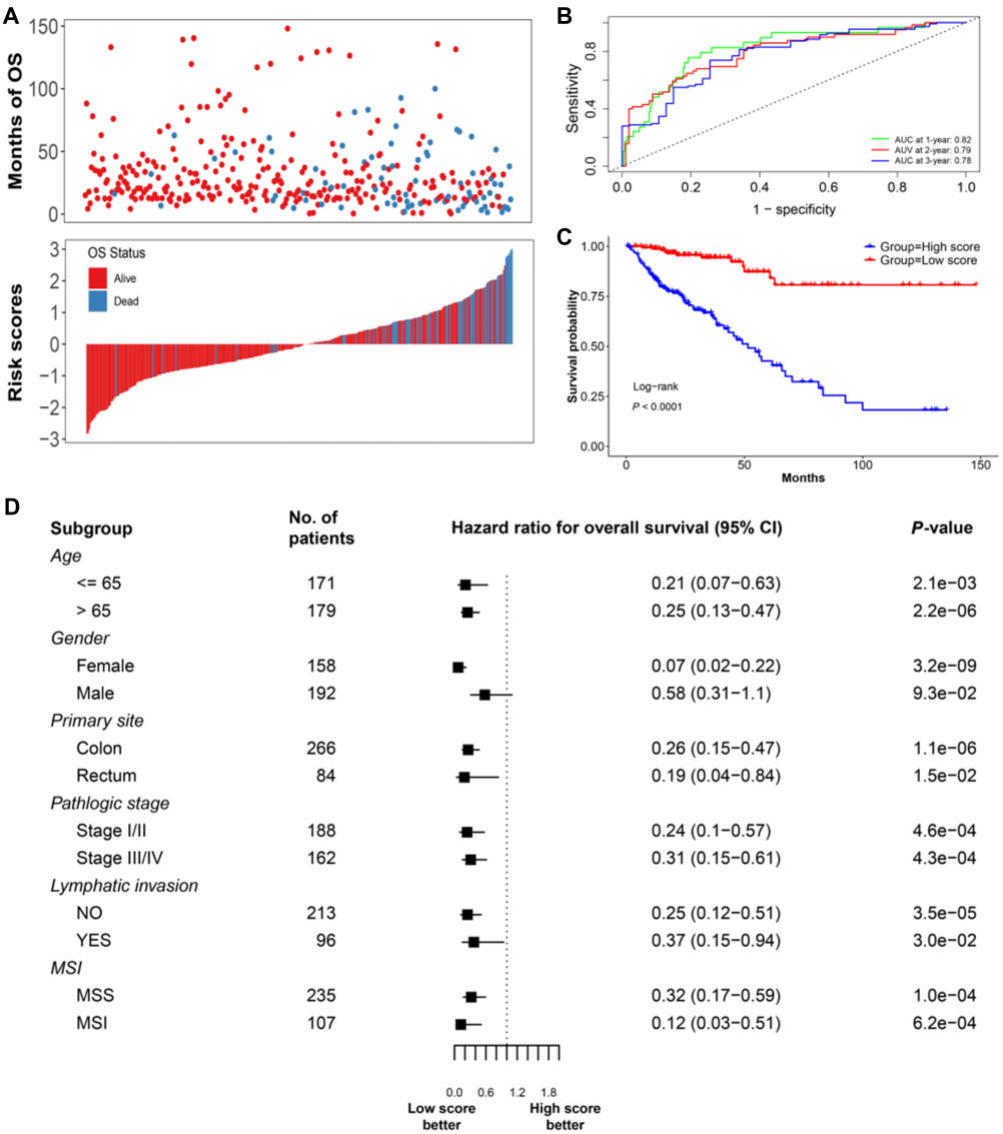
Construction of prognostic signature base on dysregulations with TCGA CRC dataset. (**A**) Scatter plot for OS time, survival status, and risk score for TCGA CRC samples. (**B**) Time-dependent ROCs for risk score at 1-, 3-, and 5-year survival. (**C**) Kaplan–Meier curves of OS between two groups cut by median risk core. *P*-value was generated from log-rank test. (**D**) Forest plots of the associations between 4-DysReg risk score and OS in various subgroups. High score: the group with risk score larger than the median; low score: the group with risk score smaller than the median.

Based on the expression data of the seven genes involved in 4-DysReg (Supplementary Figure S3) and the clinical information including age, gender, and pathological stage in TCGA CRC dataset, we built an OS cox model on the entire samples from TCGA CRC dataset. C-index of this model ran up to 0.79 (SE = 0.038). The risk scores for every sample were then calculated. The OS time, survival status, and risk score were shown in Figure 4A, indicating a high correlation between risk score and survival status. Time-dependent receiver operating characteristic (ROC) curves showed that this model represents high accuracy for OS prediction, with the area under ROC curve (AUC) at 1-, 3-, and 5-year survival reaching 0.82, 0.79, and 0.78 (Figure 4B). After that, median risk score was used to cut the samples into high and low score groups, and patients with low score displayed significantly better prognosis (hazard ratio (HR) = 0.134; 95% CI: 0.071‒0.253; *P*-value = 6.52e−10; Figure 4C). Following the same analysis procedure as in TCGA CRC dataset, the OS predictive power of 4-DysReg was also validated on independent datasets, GSE39582 and GSE17538 (Supplementary Figure S4). Besides, we checked the performance of 4-DysReg in various clinical stratification subtypes with regard to age, gender, primary site, pathological stage, lymphatic invasion, and microsatellite status. The results showed that low score group always had significantly longer survival time than high score group in almost every subtype (Figure 4D). It is noticed that patients in low score group showed significantly better prognosis in both microsatellite instability (MSI) subgroup (*n *=* *107) and microsatellite stability (MSS) subgroup (*n *=* *235) (Figure 4D). Since MSI is a mature biomarker in CRC for prognosis and for some chemotherapies (Boland and Goel, 2010), it seems that 4-DysReg has a good consistency across various subtypes for CRC prognosis prediction.

At last, cross-validation on three datasets, TCGA CRC, GSE39582, and GSE17538, indicated that C-index, 1-, 3-, and 5-year survival AUC, and log-rank test of the testing sets maintained high levels compared to the training sets (Table 1). Taken together, our signature 4-DysReg is capable of robustly predicting OS with high accuracy.

**Table 1:** Cross-validation of 4-DysReg in terms of C-indexes, AUC of time-dependent ROC at 1-, 3-, and 5-year survival, and log-rank test in three cohorts.

Cohort	C-index	AUC_1	AUC_3	AUC_5	−log10 (log-rank test *P*-value)
TCGA CRC					
Training set	0.80 (0.78‒0.81)	0.83 (0.80‒0.85)	0.80 (0.78‒0.83)	0.79 (0.76‒0.82)	7.74 (6.23‒8.81)
Testing set	0.76 (0.73‒0.78)	0.79 (0.75‒0.82)	0.76 (0.72‒0.80)	0.73 (0.69‒0.78)	4.15 (3.23‒5.01)
GSE39582					
Training set	0.71 (0.69‒0.72)	0.70 (0.65‒0.77)	0.73 (0.69‒0.82)	0.75 (0.71‒0.86)	6.97 (5.85‒8.12)
Testing set	0.67 (0.65‒0.71)	0.76 (0.72‒0.79)	0.71 (0.69‒0.74)	0.70 (0.68‒0.72)	3.41 (3.31‒4.08)
GSE17538					
Training set	0.75 (0.73‒0.77)	0.84 (0.80‒0.87)	0.78 (0.75‒0.81)	0.78 (0.76‒0.81)	3.52 (2.99‒4.23)
Testing set	0.71 (0.68‒0.75)	0.76 (0.70‒0.81)	0.69 (0.64‒0.73)	0.71 (0.65‒0.75)	1.54 (1.00‒2.08)

Data are expressed as median (first quantile‒third quantile) of 100 times of cross-validation.

### Comparison of the predictive accuracy of 4-DysReg with other CRC signatures

**Figure 5:**
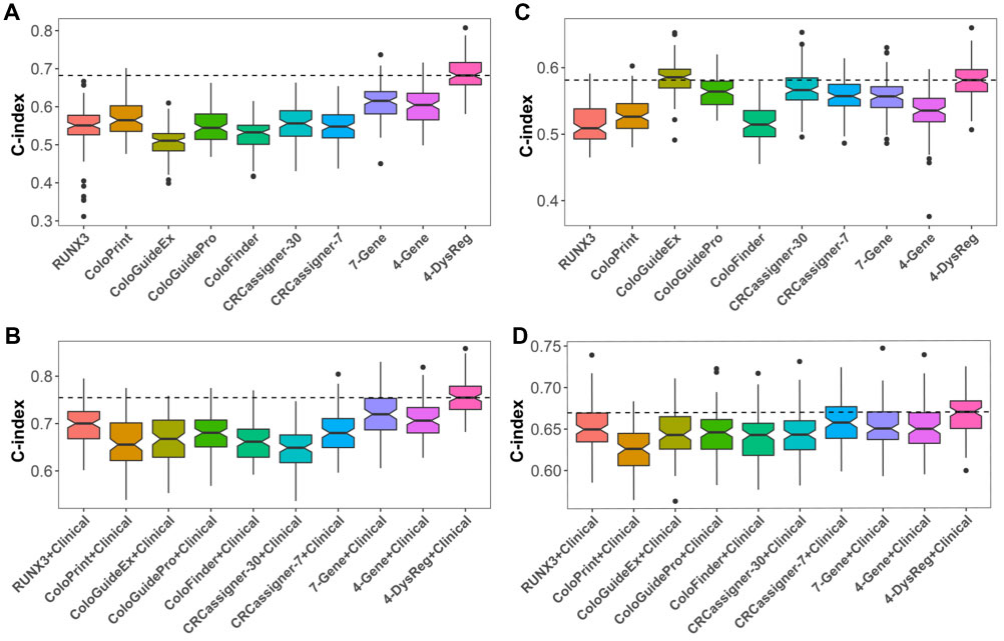
Comparison of the predictive accuracy of 4-DysReg with other CRC expression signatures. (**A**) Results in TCGA CRC dataset. (**B**) Results in TCGA CRC dataset with clinical information included. (**C**) Results in GSE39582 dataset. (**D**) Results in GSE39582 dataset with clinical information included. In this cross-validation, 60% of the samples were randomly selected as training set to fit a cox model with each signature, and the left 40% were taken as testing set to calculate C-index. Cross-validation of each signature was repeated 100 times.

We compared the predictive accuracy of 4-DysReg for OS with previously reported CRC expression signatures, including RUNX3 (Soong et al., 2009), ColoPrint (contains 18 genes) (Salazar et al., 2011), ColoGuideEx (contains 13 genes) (Agesen et al., 2012), ColoGuidePro (contains 7 genes) (Sveen et al., 2012), ColoFinder (contains 9 genes) (Shi and He, 2016), CRCassigner-30 (Sadanandam et al., 2013), CRCassigner-7 (Sadanandam et al., 2013), a 7-gene signature (Chen et al., 2017), and a 4-gene signature (Zou et al., 2015). The detailed gene information of every signature could be found in Supplementary File. In TCGA CRC dataset, our 4-DysReg signature outperformed all the others in the cross-validation (Figure 5A). With clinical information included, the accuracy of all signatures was enhanced and 4-DysReg still performed the best (Figure 5B). In GSE39582 dataset, ColoGuideEx alone displayed a slightly higher C-index than 4-DysReg (Figure 5C), while 4-DysReg surpassed ColoGuideEx when combining clinical information (Figure 5D). Taken together, these observations indicate the predictive power of 4-DysReg for prognosis.

### Predictive power of 4-DysReg for chemotherapeutic benefit

ADJC is preferred for curing CRC patients, and the guideline of ADJC is established on pathologic stage (Watanabe et al., 2018). Stage III and IV CRC patients are routinely recommended to receive ADJC. Stage II CRC patients with high risk of recurrence also consider ADJC, but the usefulness of postoperative ADJC has not been proved (Watanabe et al., 2018).

**Figure 6:**
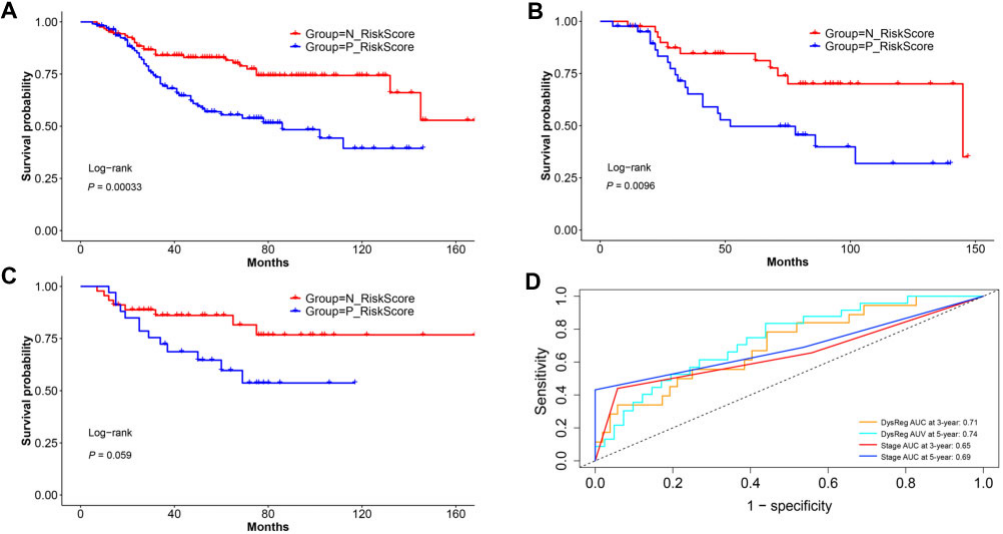
Predictive power of 4-DysReg for chemotherapeutic benefit in GSE39582. (**A**‒**C**) Kaplan‒Meier curves for OS predicted by 4-DysReg between positive and negative risk score groups in all ADJC (**A**), combined ADJC (**B**), and 5-FU (**C**). (**D**) Time-dependent ROC for combined ADJC. N_RiskScore, negative risk score predicted by 4-DysReg; P_RiskScore, positive risk score predicted by 4-DysReg. *P*-value was generated from log-rank test.

Herein, we adopted a large-scale CRC dataset GSE39582 (553 samples), with ADJC records ranging from stage II to stage IV, to explore the predictive power of 4-DysReg for chemotherapeutic benefit regardless of pathologic stage. An OS cox model was trained with the expression data of the seven genes involved in 4-DysReg on samples without ADJC (*n *=* *321), which was used to calculate risk scores of samples with ADJC (*n *=* *232) (see Supplementary File for the rationality of choosing the training set). It was shown that the sample group with negative risk score had better prognosis (HR = 0.432; 95% CI: 0.269‒0.693; Figure 6A).

Furthermore, we analyzed the predictive capability of 4-DysReg for benefit of specific chemotherapy types including 5-FU and combined ADJC (including FOLFIRI, FOLFOX, and FUFOL). Still, the samples with negative risk score basically showed better survival for both combined ADJC (*n *=* *84; HR = 0.380; 95% CI: 0.178‒0.813; Figure 6B) and 5-FU (*n *=* *79; HR = 0.437; 95% CI: 0.181‒1.055; Figure 6C). We also compared the predictive capability of 4-DysReg for chemotherapeutic benefit with previously reported signatures. Excepting CRCassigner-7 for 5-FU chemotherapy benefit, 4-DysReg performed much better when predicting chemotherapeutic benefit of all ADJC, combined ADIC, and 5-FU (Supplementary File). The accuracy of the signature for combined ADJC was evaluated by time-dependent ROC, and 3- and 5-year survival AUC reached 0.71 and 0.74, higher than AUC of model based on pathologic stage, 0.65 and 0.69 (Figure 6D).

These results demonstrate that our 4-DysReg signature possesses the predictive power for therapeutic benefit of ADJC, including only for 5-FU, only for combined ADJC, and for all ADJC, and therefore has the potential to guide ADJC regardless of pathologic stage information.

## Discussion

In the field of cancer precision medicine, a series of signatures have been built for prognosis or therapeutic benefits based on gene expression data (Chen et al., 2007; Khambata-Ford et al., 2007; Salazar et al., 2011; Sparano et al., 2019). However, current efforts are always focusing on predictive accuracy over explanatory power. Driven by the recent mechanism-driven strategies, we developed a robust gene dysregulation analysis framework by using machine learning algorithms, which is capable of robustly exploring gene dysregulations underlying carcinogenesis from high-dimensional data without ignoring cooperativity and synergy between regulators. We applied our framework on a TCGA CRC cohort and eventually developed a dysregulation-based signature with the capability of predicting prognosis and chemotherapy benefit as well. Our signature 4-DysReg showed superior performance over a series of previously reported CRC expression signatures for prognosis and chemotherapy benefit. Compared with those reported signatures generated from individual gene expression analysis based methodology (Xiong et al., 2018), 4-DysReg involves much fewer genes, and have more functional relevance in carcinogenesis in terms of dysfunctional transcriptional regulation.

It has been widely accepted that dysfunctional regulation of gene expression programs can cause a broad range of phenotypic changes, such as carcinogenesis (Lee and Young, 2013). Systematic identification of gene dysregulations based on omics data is an effective path for exploring molecular mechanisms behind phenotypic changes. Quite a few approaches have been reported to address this by measuring the change of expression correlation between gene pairs across phenotypes (de la Fuente, 2010; Ideker and Krogan, 2012; Li et al., 2016), yet in the framework of correlation analysis, this pair-based estimation inevitably overrates the contribution of an individual regulator to its target. In order to simultaneously consider the contributions of multiple regulators to transcriptional regulations, even the cooperative and synergistic effect between multiple regulators (Lambert et al., 2018), we have previously developed dysregulation analysis methods based on multivariate regression, which were applied to cancer and generated carcinogenesis-relevant biomarkers (Cao et al., 2015; Li et al., 2017b). However, the methodology is far from robust when dealing with high-dimensional transcriptome data for dysregulation analysis on a systematic level.

In the present dysregulation analysis framework, the cooperative and synergistic effect between regulators could be fully considered in a robust way. First, conditional GRNs are constructed by selecting important TFs for target expression with feature selection algorithm *Boruta*, in which the relative importance of all candidate TFs are estimated simultaneously (Kursa and Rudnicki, 2010); secondly, regulatory intensities and its CIs of remaining TFs for target expression are quantified with de-biased LASSO (Javanmard and Montanari, 2014), where the expression of the target is determined by the combined regulatory effect of its all possible regulators, which naturally considers the cooperativity and synergy between TFs. Besides, we identify gene dysregulations according to three factors including differential regulation, differential expression of target, and the consistency between differential regulation and differential expression. In this way, our dysregulation analysis framework is robust in dealing with high-dimensional transcriptome data and endowed with explanatory power by combining biological principles and machine learning algorithms with interpretability.

The explanatory signature 4-DysReg built in this study consists of four dysregulations including RUNX3→GPR15, RUNX3→P2RY8, SNAI3→TLR7, and ATOH1→SIGLEC1. The 4-DysReg showed good performance in predicting prognosis and therapeutic benefit of ADJC in CRC patients. Attributed to dysregulation analysis, 4-DysReg has sufficient interpretability in terms of dysfunctional transcriptional regulation and molecular functions. As expected, the seven genes involved in 4-DysReg have been reported to participate in diverse processes related to carcinogenesis. RUNX3 is a member of the runt domain-containing family of TFs, and participates in diverse processes including proliferation, differentiation, cell lineage specification, apoptosis, and DNA repair (Ito et al., 2015; Bae et al., 2019). In CRC, RUNX3 is taken as a tumor suppressor since its methylation is a significant risk factor for tumor development and high nuclear expression of RUNX3 is associated with better survival (Weisenberger et al., 2006; Soong et al., 2009). It is intriguing that RUNX3 demonstrates both tumor-suppressive and oncogenic activities in multiple solid tumors (Ito et al., 2015). Still intriguingly, GPR15, an orphan G-protein-linked receptor, is recently reported to mediate T-cells localization to colon when expressed on cell surface of T cells (Habtezion et al., 2016), while play a supporting role in anti-inflammatory process (Pan et al., 2017) when expressed on cell surface of vascular endothelial cells. P2RY8, an orphan G protein-coupled receptor like GPR15, is a key regulator for affinity maturation of B cells in germinal centers (Muppidi et al., 2014). SNAI3 was identified as an invasion-related marker (Puisieux et al., 2014), and also reported to play a key role in differentiation of lymphoid cells and myeloid cells (Dahlem et al., 2012). TLR7, a dual receptor for guanosine and uridine-containing ssRNA in innate immunity, could perform tumor-suppressive activity by mediating the activation of NFκB and inducing proinflammatory cytokines (Schon and Schon, 2008). This gene has been recognized as a hot target for tumor targeted immunotherapy and cancer vaccines (Schon and Schon, 2008; Lynn et al., 2020). ATOH1 is a master TF for regeneration and differentiation of intestinal epithelial cells (Ishibashi et al., 2018). SIGLEC1, also named as CD169, could function as a facilitator of the recognition and internalization of sialic acid decorated apoptotic bodies and exosomes derived from tumors, which potentially contributes to both attenuation and facilitation of anti-tumor immunity (Fraschilla and Pillai, 2017). It is appealing that several genes among 4-Dysreg possess immune-related functions, including RUNX3, GPR15, P2RY8, SNAI3, TLR7, and SIGLEC1. We have actually observed the positive correlation between the expression of these genes and the abundance of eight immune cell types within tumor tissue (Supplementary File and Supplementary Figure S5), which provides functional interpretability of 4-DysReg from the viewpoint of cellular functions among tissue.

It is noted that some signature genes, e.g. RUNX3, display opposite functions in different studies. Although RUNX3 was reported to be a tumor suppressor in CRC (Weisenberger et al., 2006; Soong et al., 2009), in TCGA CRC dataset, RUNX3 expression displays weak difference between normal and cancer (Supplementary Figure S6) and weak correlation with prognosis (HR = 1.125, 95% CI: 0.965‒1.312). In line with previous observations, it is suggested that cytoplasmic localization of RUNX3 is a main mode of RUNX3 inactivation (Soong et al., 2009; Ito et al., 2015), which explains why only RUNX3 displays limited prediction accuracy for prognosis (Figure 5A and C). Benefitting from gene dysregulation analysis, we obtained functional relevance of RUNX3 from its one target, GPR15. Still, in TCGA CRC dataset, the expression of GPR15 significantly decreases in cancer (Supplementary Figure S6) and is significantly correlated with good prognosis (HR = 0.735, 95% CI: 0.621‒0.870). This observation supports the tumor-suppressive activity of GPR15, consistent with its immunomodulatory role (Habtezion et al., 2016) and its therapeutic potential proposed very recently (Namkoong et al., 2019). According to gene dysregulation analysis, the regulatory intensity of RUNX3→GPR15 is decreased from normal to cancer (Figure 2A), which suggests that RUNX3 at least partly contributes to the high expression of GPR15 in normal. This explains the anti-cancer activity of RUNX3 in CRC. In this case, our dysregulation analysis links the two individual genes in the gene transcriptional regulation scenario, characterizes the regulation intensity under specific condition, and implies the role of dysregulation of RUNX3→GPR15 in CRC carcinogenesis, which is obviously worthy of further in-depth investigation.

Beyond this, we would bring up again what we proposed in 2011 that the attention to correlation change will help to explore subtle mechanisms involved in tuning of molecular balances between opposite factors (Yu et al., 2011). Quite a lot of efforts have actually been devoted to elucidating the mechanism of the selectivity of cell fates, or, the tip of balance of molecular events. For example, the activation of p53 induced by DNA damage would lead to cell cycle arrest allowing for DNA repair if the damage is mild, or irreparably trigger apoptosis if the damage is severe (Kastenhuber and Lowe, 2017; Levine, 2019). A common issue is, when a key molecular component (p53 in the above example) was identified to be associated with diverse events (DNA repair and apoptosis) in response to a common signal (genotoxic stress), how to determine the mechanism by which the key component determines which genes to turn on or off from a plethora of partner proteins to achieve the desirable cellular outcome. As 10 years ago, we believe differential regulation analysis, or dysregulation analysis, could provide a promising solution to address this issue. In the RUNX3→GPR15 case, we adopted dysregulation relationship between RUNX3 and GPR15 to confirm the anti-cancer function of RUNX3 in CRC. Meanwhile, there might be unknown partners of RUNX3 that confer RUNX3 oncogenic activity in other conditions. This implication is not limited to RUNX3 and GPR15, but also could be extended to other genes or regulations in the context of gene transcriptional dysregulation.

Despite the promising results, still there is a large room for improvement in our approach. First, our reference GRN is derived from the predicting TF binding site in 1000 bp sequence ahead transcription start site, which is one of the most general manner used to build reference GRN at present (Lambert et al., 2018). It should be noted that promoter sequence could be longer for some genes, and distant enhancers also play important role in gene transcription (Schoenfelder and Fraser, 2019). Additionally, the tool of predictive TF binding site used in this work, FIMO, is based on the similarity between TF motif and DNA sequence (Grant et al., 2011). Besides DNA sequence, the binding is also influenced by other features, such as chromosome accessibility, histone modification, DNA methylation, TF post-transcriptional modification and TF combination (Lambert et al., 2018). How to build a reference GRN with high reliability for TF–DNA binding is a hot topic in the area. Secondly, gene dysregulations in the present study were identified with only 32 paired samples. We believe a larger dataset could enhance the performance of dysregulation analysis and prognostic signature. At last but not least, based on the effectiveness of 4-DysReg proved by the present study, we will include a baseline dataset with large sample size and build a prognostic model with seven genes involved in 4-DysReg in future.

In summary, we presented a gene dysregulation analysis framework, which is capable of exploring gene dysregulations underlying carcinogenesis from high-dimensional data with cooperativity and synergy of multiple regulators and several other transcriptional regulation rules taken into consideration. The framework was applied to CRC dataset, and the signature 4-DysReg was built based the identified CRC-related dysregulations. 4-DysReg has the capability of predicting prognosis and chemotherapy benefit, and the potential of explaining carcinogenesis in terms of dysfunctional transcriptional regulation. It is our belief that the gene dysregulation analysis framework will help to elucidate systematic mechanisms of carcinogenesis and develop signatures with high predictive accuracy and mechanistic interpretability in clinical application.

## Materials and methods

### Collecting and preprocessing expression data

TCGA CRC mRNA data and clinical data were downloaded from UCSC Xena (Vivian et al., 2017), and 380 primary tumor samples and 51 adjacent normal samples were selected. The abundance of mRNA was determined as transcripts per million (TPM). The TPM values <1 were treated as missing values. For a certain gene, when the number of missing values was >20% of total sample size, the gene was deleted. The remaining missing data were filled in with k-nearest neighbor method (Troyanskaya et al., 2001). The expression data were log2 transformed. Among them, 32 pairs of matched tumor and adjacent normal samples were used to construct conditional GRNs and identify gene dysregulations; 350 primary tumor samples with OS, RFS, age, gender, and pathological stage information were used in prognostic analysis.

Two expression datasets with clinical data including OS, RFS, age, gender, and pathological stage, GSE39582 (Marisa et al., 2013) and GSE17538 (Smith et al., 2010), were downloaded from GEO (http://www.ncbi.nlm.nih.gov/geo/). GSE39582 contains 566 CRC tumor samples and 19 normal samples. GSE17538 contains 238 CRC tumor samples. Both datasets are based on GPL570 platform. The probes matching to multiple genes were deleted. When >1 probe could be mapped to the same gene, the probe with the highest value was taken to represent the expression level of the gene. The way of handling missing data was the same as that for TCGA data. The expression data were log2 transformed. Quantile method was adopted to normalize data between samples (Bolstad et al., 2003). A total of 553 tumor samples in GSE39582 and 200 tumor samples in GSE17538 with sufficient clinical information were used in prognostic analysis.

### Constructing reference GRN

TF and the ‘best’ motif data were accessed from HumanTF database (Lambert et al., 2018). The 1000 bp upstream sequences from transcription start site of all coding genes were acquired by biomaRt (http://www.biomart.org/) and regarded as promoter regions. The promoter region of each gene was scanned with FIMO (Grant et al., 2011) with *P*-value <1e−04. For each TF, the threshold that the probability of making at least one false discovery was <0.01 was adopted to perform multi-hypothesis test correction to select its targets. If a TF had >5000 targets, only the top 5000 targets ranked by FIMO score were kept (Grant et al., 2011). The remaining TF‒target relationships, or candidate TF‒targets, composed the reference GRN (Figure 1A), which included 1083 TFs and 1773407 relationships.

### Constructing conditional GRNs

Based on expression data under a specific condition, TF‒target relationships in reference GRN were filtered by using a feature selection method *Boruta*, a wrapper around random forest algorithm (Kursa and Rudnicki, 2010). For each target, the expression values of its candidate TFs in reference GRN were regarded as original features, and the target’s expression was regarded as response variable. By shuffling candidate TFs, *Boruta* generated shadow features, iteratively estimated the importance of every TF, and removed the TF less relevant to its target. In this way, those conceptual links which did not perform functions under specific condition were removed from reference GRN, and the remaining TF‒target relationships formed conditional GRNs (Figure 1B), i.e. normal GRN and cancer GRN in the present study.

### Identifying gene dysregulations

Gene dysregulations were identified by integrating three standards. Firstly, the regulatory intensity should be significantly different between conditions; secondly, target should be differentially expressed; lastly, the change direction of regulatory intensity should be consistent with the change direction of target’s expression between conditions (Figure 1C).

Since the de-biased LASSO method is able to robustly obtain the estimation and the covariance of regression coefficients for high-dimensional regression (Javanmard and Montanari, 2014), it was adopted to estimate the regression coefficients and their CIs of every upstream TF of a certain target, with regression coefficients and CIs taken as regulatory intensities and their ranges. For each regulation, if 95% CIs of the regression coefficient have no overlap between normal and cancer conditions, the regulatory intensity could be regarded as significantly differential.

For target expression change, we carried out differential expression analysis by using limma (Ritchie et al., 2015) with the cutoff of |logFC| > 1 and *P*_adj_ < 0.05 on log2-transformed expression data. DEGs were identified and their change directions, say activation of inhibition, were recorded.

Eventually, for a certain regulation, the TF whose regulatory intensity change was consistent with its target’s expression change from one condition to another was considered to play key role in controlling target’s expression. And the regulations with consistent change of regulatory intensity and target’s expression were kept in the following analysis.

### Checking prognostic effect of dysregulations

For each gene dysregulation, four types of cox proportional hazard models were constructed in TCGA CRC dataset by using R package survival (https://cran.r-project.org/web/packages/survival/), fitted with (i) two genes’ expression data adjuvant with clinical information including age, gender, and pathological stage for OS, (ii) two genes’ expression data for OS, (iii) two genes’ expression data adjuvant with clinical information including age, gender, and pathological stage for RFS, and (iv) two genes’ expression data for RFS. For each cox model, C-index was calculated to measure the prediction accuracy. Meanwhile, four groups of controls were set with the following procedures, each containing the same number of randomly selected gene pairs as the identified gene dysregulations: (i) two genes were randomly selected from the gene list of preprocessed expression data; (ii) one DEG and one non-differentially expressed gene (non-DEG) were randomly selected from the gene list of preprocessed expression data; (iii) one regulation was randomly selected from the reference GRN; and (iv) one regulation whose target is DEG was randomly selected from the reference GRN. Still, four types of cox models were built for each random pair, and C-index was calculated for each model. At last, for each type of cox models, Wilcox test was used to check whether the C-indexes from the identified dysregulations were significantly larger than those from random pairs (one-way Wilcox test), i.e. whether the accuracy of cox models fitted by the identified dysregulations as a whole was higher than by controls. This process was repeated 100 times. The prognostic effect of the identified dysregulations was also validated in GSE39582 and GSE17538.

### Building prognostic signature

Among the OS cox models fitted with gene expression data of every dysregulation adjuvant with clinical information in TCGA CRC dataset as described above, the model with the largest C-index was taken as the primary one, and then a greedy strategy was adopted to exclusively add every other candidate dysregulation to the primary model. For each new model, 60% of the samples were randomly selected as training set to construct a cox model, and the left 40% of the samples were taken as testing set to calculate C-index of the cox model. This cross-validation was repeated 100 times and the median C-index was assigned to the new model. For every iteration, the model was finally updated by integrating one more dysregulation which led to the highest C-index. The iteration ended up with C-index of the updated model being steady (ΔC-index < 0.001). The dysregulations involved in the final model were taken as prognostic signature.

Risk score of each sample was calculated by genes in the prognostic signature and clinical factors including age, gender, and histological type with cox multivariate regression among all samples in the dataset. Samples were divided into two groups with the median of risk scores, and Kaplan‒Meier (KM) survival analysis was then carried out to evaluate the difference in survival time between the two groups. The accuracy of the risk score for predicting survival was evaluated by C-index and AUC with *timeROC* (Blanche et al., 2013). These analyses were also conducted in two independent datasets.

The prognostic signature was further validated following a cross-validation procedure. That is, 60% of the samples were randomly selected as training set to fit a cox model as described above, and the left 40% were taken as testing set to calculate C-index, AUC at 1-, 3-, 5-year OS, and significance of KM survival curves. The cross-validation was repeated 100 times. This cross-validation procedure was respectively conducted in TCGA CRC and two independent datasets.

### Enrichment analysis

Pathway over-representation analysis was implemented on ConcensusPathDB (Herwig et al., 2016), which is a comprehensive collection of human molecular interaction data integrating different public repositories. The pathway databases used in ConcensusPathDB include KEGG, Reactome, WikiPathways, Biocarta, and PharmGKB. Over-representation pathways were extracted with the threshold that pathway had at least five genes overlapped with input genes and *P*-value was <0.05.

GSEA was performed with GSEA3.0 software (Subramanian et al., 2005). The gene set database ‘c5.bp.v6.2.symbols.gmt [Gene ontology]’ was used to determine the enrichment of gene sets.

## Supplementary material

Supplementary material is available at *Journal of Molecular Cell Biology* online.

## Supplementary Material

mjaa041_Supplementary_Data

## References

[mjaa041-B1] Agesen T.H. , Sveen A., Merok M.A., et al (2012). ColoGuideEx: a robust gene classifier specific for stage II colorectal cancer prognosis. Gut 61, 1560–1567. 22213796 10.1136/gutjnl-2011-301179

[mjaa041-B2] Bae S.C. , Kolinjivadi A.M., Ito Y. (2019). Functional relationship between p53 and RUNX proteins. J. Mol. Cell Biol. 11, 224–230. 30535344 10.1093/jmcb/mjy076 PMC6478125

[mjaa041-B3] Barzel B. , Barabasi A.L. (2013). Network link prediction by global silencing of indirect correlations. Nat. Biotechnol. 31, 720–725. 23851447 10.1038/nbt.2601 PMC3740009

[mjaa041-B4] Blanche P. , Dartigues J.F., Jacqmin-Gadda H. (2013). Estimating and comparing time-dependent areas under receiver operating characteristic curves for censored event times with competing risks. Stat. Med. 32, 5381–5397. 24027076 10.1002/sim.5958

[mjaa041-B5] Boland C.R. , Goel A. (2010). Microsatellite instability in colorectal cancer. Gastroenterology 138, 2073–2087.e3. 20420947 10.1053/j.gastro.2009.12.064 PMC3037515

[mjaa041-B6] Bolstad B.M. , Irizarry R.A., Astrand M., et al (2003). A comparison of normalization methods for high density oligonucleotide array data based on variance and bias. Bioinformatics 19, 185–193. 12538238 10.1093/bioinformatics/19.2.185

[mjaa041-B7] Bray F. , Ferlay J., Soerjomataram I., et al (2018). Global cancer statistics 2018: GLOBOCAN estimates of incidence and mortality worldwide for 36 cancers in 185 countries. CA Cancer J. Clin. 68, 394–424. 30207593 10.3322/caac.21492

[mjaa041-B8] Cao M.S. , Liu B.Y., Dai W.T., et al (2015). Differential network analysis reveals dysfunctional regulatory networks in gastric carcinogenesis. Am. J. Cancer Res. 5, 2605–2625. 26609471 PMC4633893

[mjaa041-B9] Chen H. , Sun X., Ge W., et al (2017). A seven-gene signature predicts overall survival of patients with colorectal cancer. Oncotarget 8, 95054–95065. 29221110 10.18632/oncotarget.10982 PMC5707004

[mjaa041-B10] Chen H.Y. , Yu S.L., Chen C.H., et al (2007). A five-gene signature and clinical outcome in non-small-cell lung cancer. N. Engl. J. Med. 356, 11–20. 17202451 10.1056/NEJMoa060096

[mjaa041-B11] Dahlem T. , Cho S., Spangrude G.J., et al (2012). Overexpression of Snai3 suppresses lymphoid- and enhances myeloid-cell differentiation. Eur. J. Immunol. 42, 1038–1043. 22531927 10.1002/eji.201142193 PMC3369572

[mjaa041-B12] Dai W. , Li Q., Liu B.Y., et al (2018). Differential networking meta-analysis of gastric cancer across Asian and American racial groups. BMC Syst. Biol. 12, 51. 29745833 10.1186/s12918-018-0564-z PMC5998874

[mjaa041-B13] de la Fuente A. (2010). From ‘differential expression’ to ‘differential networking’—identification of dysfunctional regulatory networks in diseases. Trends Genet. 26, 326–333. 20570387 10.1016/j.tig.2010.05.001

[mjaa041-B14] Fraschilla I. , Pillai S. (2017). Viewing Siglecs through the lens of tumor immunology. Immunol. Rev. 276, 178–191. 28258691 10.1111/imr.12526 PMC5860639

[mjaa041-B15] Grant C.E. , Bailey T.L., Noble W.S. (2011). FIMO: scanning for occurrences of a given motif. Bioinformatics 27, 1017–1018. 21330290 10.1093/bioinformatics/btr064 PMC3065696

[mjaa041-B16] Habtezion A. , Nguyen L.P., Hadeiba H., et al (2016). Leukocyte trafficking to the small intestine and colon. Gastroenterology 150, 340–354. 26551552 10.1053/j.gastro.2015.10.046 PMC4758453

[mjaa041-B17] Herwig R. , Hardt C., Lienhard M., et al (2016). Analyzing and interpreting genome data at the network level with ConsensusPathDB. Nat. Protoc. 11, 1889–1907. 27606777 10.1038/nprot.2016.117

[mjaa041-B18] Ideker T. , Krogan N.J. (2012). Differential network biology. Mol. Syst. Biol. 8, 565. 22252388 10.1038/msb.2011.99 PMC3296360

[mjaa041-B19] Ishibashi F. , Shimizu H., Nakata T., et al (2018). Contribution of ATOH1^+^ cells to the homeostasis, repair, and tumorigenesis of the colonic epithelium. Stem Cell Rep. 10, 27–42. 10.1016/j.stemcr.2017.11.006 PMC5768891 29233556

[mjaa041-B20] Ito Y. , Bae S.C., Chuang L.S. (2015). The RUNX family: developmental regulators in cancer. Nat. Rev. Cancer 15, 81–95. 25592647 10.1038/nrc3877

[mjaa041-B21] Javanmard A. , Montanari A. (2014). Confidence intervals and hypothesis testing for high-dimensional regression. J. Mach. Learn. Res. 15, 2869–2909.

[mjaa041-B22] Kastenhuber E.R. , Lowe S.W. (2017). Putting p53 in context. Cell 170, 1062–1078. 28886379 10.1016/j.cell.2017.08.028 PMC5743327

[mjaa041-B23] Khambata-Ford S. , Garrett C.R., Meropol N.J., et al (2007). Expression of epiregulin and amphiregulin and K-ras mutation status predict disease control in metastatic colorectal cancer patients treated with cetuximab. J. Clin. Oncol. 25, 3230–3237. 17664471 10.1200/JCO.2006.10.5437

[mjaa041-B24] Kursa M.B. , Rudnicki W.R. (2010). Feature selection with the Boruta package. J. Stat. Softw. 36, 13.

[mjaa041-B25] Lambert S.A. , Jolma A., Campitelli L.F., et al (2018). The human transcription factors. Cell 172, 650–665. 29425488 10.1016/j.cell.2018.01.029 PMC12908702

[mjaa041-B26] Lee T.I. , Young R.A. (2013). Transcriptional regulation and its misregulation in disease. Cell 152, 1237–1251. 23498934 10.1016/j.cell.2013.02.014 PMC3640494

[mjaa041-B27] Levine A.J. (2019). The many faces of p53: something for everyone. J. Mol. Cell Biol. 11, 524–530. 30925588 10.1093/jmcb/mjz026 PMC6736316

[mjaa041-B28] Li J. , Li Y.X., Li Y.Y. (2016). Differential regulatory analysis based on coexpression network in cancer research. BioMed Res. Int. 2016, 4241293. 27597964 10.1155/2016/4241293 PMC4997028

[mjaa041-B29] Li J. , Wu S., Yang L., et al (2017a). Integrated differential regulatory analysis reveals a novel prognostic 36-gene signature for gastric cancer in Asian population. Comb. Chem. High Throughput Screen. 20, 174–181. 28124598 10.2174/1386207320666170117121543

[mjaa041-B30] Li Q. , Li J., Dai W., et al (2017b). Differential regulation analysis reveals dysfunctional regulatory mechanism involving transcription factors and microRNAs in gastric carcinogenesis. Artif. Intell. Med. 77, 12–22. 28545608 10.1016/j.artmed.2017.02.006

[mjaa041-B31] Liu B.H. , Yu H., Tu K., et al (2010). DCGL: an R package for identifying differentially coexpressed genes and links from gene expression microarray data. Bioinformatics 26, 2637–2638. 20801914 10.1093/bioinformatics/btq471 PMC2951087

[mjaa041-B32] Lu Y. , Fang Z., Li M., et al (2019). Dynamic edge-based biomarker non-invasively predicts hepatocellular carcinoma with hepatitis B virus infection for individual patients based on blood testing. J. Mol. Cell Biol. 11, 665–677. 30925583 10.1093/jmcb/mjz025 PMC6788726

[mjaa041-B33] Lynn G.M. , Sedlik C., Baharom F., et al (2020). Peptide-TLR-7/8a conjugate vaccines chemically programmed for nanoparticle self-assembly enhance CD8 T-cell immunity to tumor antigens. Nat. Biotechnol. 38, 320–332. 31932728 10.1038/s41587-019-0390-x PMC7065950

[mjaa041-B34] Marisa L. , de Reynies A., Duval A., et al (2013). Gene expression classification of colon cancer into molecular subtypes: characterization, validation, and prognostic value. PLoS Med. 10, e1001453. 23700391 10.1371/journal.pmed.1001453 PMC3660251

[mjaa041-B35] Muppidi J.R. , Schmitz R., Green J.A., et al (2014). Loss of signalling via Gα13 in germinal centre B-cell-derived lymphoma. Nature 516, 254–258. 25274307 10.1038/nature13765 PMC4267955

[mjaa041-B36] Namkoong H. , Lee B., Koh S.-J., et al (2019). Mo1781—role of G protein-coupled receptor 15 in colon cancer. Gastroenterology 156, S-836.

[mjaa041-B37] Pan B. , Wang X., Kojima S., et al (2017). The fifth epidermal growth factor like region of thrombomodulin alleviates LPS-induced sepsis through interacting with GPR15. Thromb. Haemost. 117, 570–579. 28078348 10.1160/TH16-10-0762

[mjaa041-B38] Puisieux A. , Brabletz T., Caramel J. (2014). Oncogenic roles of EMT-inducing transcription factors. Nat. Cell Biol. 16, 488–494. 24875735 10.1038/ncb2976

[mjaa041-B39] Ritchie M.E. , Phipson B., Wu D., et al (2015). limma powers differential expression analyses for RNA-sequencing and microarray studies. Nucleic Acids Res. 43, e47. 25605792 10.1093/nar/gkv007 PMC4402510

[mjaa041-B40] Robinson W.H. , Lindstrom T.M., Cheung R.K., et al (2013). Mechanistic biomarkers for clinical decision making in rheumatic diseases. Nat. Rev. Rheumatol. 9, 267–276. 23419428 10.1038/nrrheum.2013.14 PMC3673766

[mjaa041-B41] Sadanandam A. , Lyssiotis C.A., Homicsko K., et al (2013). A colorectal cancer classification system that associates cellular phenotype and responses to therapy. Nat. Med. 19, 619–625. 23584089 10.1038/nm.3175 PMC3774607

[mjaa041-B42] Salazar R. , Roepman P., Capella G., et al (2011). Gene expression signature to improve prognosis prediction of stage II and III colorectal cancer. J. Clin. Oncol. 29, 17–24. 21098318 10.1200/JCO.2010.30.1077

[mjaa041-B43] Schoenfelder S. , Fraser P. (2019). Long-range enhancer–promoter contacts in gene expression control. Nat. Rev. Genet. 20, 437–455. 31086298 10.1038/s41576-019-0128-0

[mjaa041-B44] Schon M.P. , Schon M. (2008). TLR7 and TLR8 as targets in cancer therapy. Oncogene 27, 190–199. 18176600 10.1038/sj.onc.1210913

[mjaa041-B45] Schwaederle M. , Zhao M., Lee J.J., et al (2015). Impact of precision medicine in diverse cancers: a meta-analysis of phase II clinical trials. J. Clin. Oncol. 33, 3817–3825. 26304871 10.1200/JCO.2015.61.5997 PMC4737863

[mjaa041-B46] Shi M. , He J. (2016). ColoFinder: a prognostic 9-gene signature improves prognosis for 871 stage II and III colorectal cancer patients. PeerJ. 4, e1804. 26989635 10.7717/peerj.1804 PMC4793313

[mjaa041-B47] Smith J.J. , Deane N.G., Wu F., et al (2010). Experimentally derived metastasis gene expression profile predicts recurrence and death in patients with colon cancer. Gastroenterology 138, 958–968. 19914252 10.1053/j.gastro.2009.11.005 PMC3388775

[mjaa041-B48] Soong R. , Shah N., Peh B.K., et al (2009). The expression of RUNX3 in colorectal cancer is associated with disease stage and patient outcome. Br. J. Cancer 100, 676–679. 19223906 10.1038/sj.bjc.6604899 PMC2653772

[mjaa041-B49] Sparano J.A. , Gray R.J., Ravdin P.M., et al (2019). Clinical and genomic risk to guide the use of adjuvant therapy for breast cancer. N. Engl. J. Med. 380, 2395–2405. 31157962 10.1056/NEJMoa1904819 PMC6709671

[mjaa041-B50] Subramanian A. , Tamayo P., Mootha V.K., et al (2005). Gene set enrichment analysis: a knowledge-based approach for interpreting genome-wide expression profiles. Proc. Natl Acad. Sci. USA 102, 15545–15550. 16199517 10.1073/pnas.0506580102 PMC1239896

[mjaa041-B51] Sveen A. , Agesen T.H., Nesbakken A., et al (2012). ColoGuidePro: a prognostic 7-gene expression signature for stage III colorectal cancer patients. Clin. Cancer Res. 18, 6001–6010. 22991413 10.1158/1078-0432.CCR-11-3302

[mjaa041-B52] Taylor I.W. , Linding R., Warde-Farley D., et al (2009). Dynamic modularity in protein interaction networks predicts breast cancer outcome. Nat. Biotechnol. 27, 199–204. 19182785 10.1038/nbt.1522

[mjaa041-B53] Topalian S.L. , Taube J.M., Anders R.A., et al (2016). Mechanism-driven biomarkers to guide immune checkpoint blockade in cancer therapy. Nat. Rev. Cancer 16, 275–287. 27079802 10.1038/nrc.2016.36 PMC5381938

[mjaa041-B54] Troyanskaya O. , Cantor M., Sherlock G., et al (2001). Missing value estimation methods for DNA microarrays. Bioinformatics 17, 520–525. 11395428 10.1093/bioinformatics/17.6.520

[mjaa041-B55] Vargas A.J. , Harris C.C. (2016). Biomarker development in the precision medicine era: lung cancer as a case study. Nat. Rev. Cancer 16, 525–537. 27388699 10.1038/nrc.2016.56 PMC6662593

[mjaa041-B56] Vivian J. , Rao A.A., Nothaft F.A., et al (2017). Toil enables reproducible, open source, big biomedical data analyses. Nat. Biotechnol. 35, 314–316. 28398314 10.1038/nbt.3772 PMC5546205

[mjaa041-B57] Walther A. , Johnstone E., Swanton C., et al (2009). Genetic prognostic and predictive markers in colorectal cancer. Nat. Rev. Cancer 9, 489–499. 19536109 10.1038/nrc2645

[mjaa041-B58] Watanabe T. , Muro K., Ajioka Y., et al (2018). Japanese Society for Cancer of the Colon and Rectum (JSCCR) guidelines 2016 for the treatment of colorectal cancer. Int. J. Clin. Oncol. 23, 1–34. 28349281 10.1007/s10147-017-1101-6 PMC5809573

[mjaa041-B59] Weisenberger D.J. , Siegmund K.D., Campan M., et al (2006). CpG island methylator phenotype underlies sporadic microsatellite instability and is tightly associated with BRAF mutation in colorectal cancer. Nat. Genet. 38, 787–793. 16804544 10.1038/ng1834

[mjaa041-B60] Wu S. , Li J., Cao M., et al (2016). A novel integrated gene coexpression analysis approach reveals a prognostic three-transcription-factor signature for glioma molecular subtypes. BMC Syst. Biol. 10(Suppl 3), 71. 27586240 10.1186/s12918-016-0315-y PMC5009532

[mjaa041-B61] Xiong Y. , You W., Hou M., et al (2018). Nomogram integrating genomics with clinicopathologic features improves prognosis prediction for colorectal cancer. Mol. Cancer Res. 16, 1373–1384. 29784666 10.1158/1541-7786.MCR-18-0063

[mjaa041-B62] Yang J. , Yu H., Liu B.H., et al (2013). DCGL v2.0: an R package for unveiling differential regulation from differential co-expression. PLoS One 8, e79729. 24278165 10.1371/journal.pone.0079729 PMC3835854

[mjaa041-B63] Yu H. , Liu B.H., Ye Z.Q., et al (2011). Link-based quantitative methods to identify differentially coexpressed genes and gene pairs. BMC Bioinformatics 12, 315. 21806838 10.1186/1471-2105-12-315 PMC3199761

[mjaa041-B64] Zickenrott S. , Angarica V.E., Upadhyaya B.B., et al (2016). Prediction of disease‒gene‒drug relationships following a differential network analysis. Cell Death Dis. 7, e2040. 26775695 10.1038/cddis.2015.393 PMC4816176

[mjaa041-B65] Zou M. , Zhang P.J., Wen X.Y., et al (2015). A novel mixed integer programming for multi-biomarker panel identification by distinguishing malignant from benign colorectal tumors. Methods 83, 3–17. 25980368 10.1016/j.ymeth.2015.05.011

