# Supplementary material for: Gene dysregulation analysis builds a mechanistic signature for prognosis and therapeutic benefit in colorectal cancer
Source: J Mol Cell Biol. 2020 Jul 27;12(11):881–93. doi: 10.1093/jmcb/mjaa041 (PMC7883816; doi:10.1093/jmcb/mjaa041)
Supplement: mjaa041_Supplementary_Data [file mjaa041_supplementary_data.zip › Supplementary material.pdf]

# Supplementary material

## Gene dysregulation analysis builds a mechanistic signature for prognosis and therapeutic benefit in colorectal cancer

Quanxue Li[1,2], Wentao Dai[2, 4], Jixiang Liu[2, 4], Yi-Xue Li[1,2,3,4,*], and Yuan-Yuan Li[2, 4,*]

[1] School of Biotechnology, East China University of Science and Technology, Shanghai 200237, China

[2] Shanghai Center for Bioinformation Technology, Shanghai 201203, China

[3] Bio-Med Big Data Center, CAS Key Laboratory of Computational Biology, CAS-MPG Partner Institute for Computational Biology, Shanghai Institute of Nutrition and Health, University of Chinese Academy of Sciences, Chinese Academy of Sciences, Shanghai 200031, China

[4] Shanghai Engineering Research Center of Pharmaceutical Translation & Shanghai Industrial Technology Institute, Shanghai Industrial Technology Institute, Shanghai 201203, China

* Correspondence to: Yuan-Yuan Li, E-mail: yyli@scbit.org; Yi-Xue Li, E-mail: yxli@sibs.ac.cn; Tel: 86-21-20283720; Fax: 86-21-20283780

## Supplementary File

### Validate the quantifing method of regulatory intensity

In order to validate the effectiveness of the quantitative method of regulatory intensity, we defined a differential regulation network (DRN), the edges of which were weighted by the difference of regulatory intensity across normal and cancer. COSMIC cancer genes (Sondka et al., 2018) showed significantly higher weights than non-cancer genes in the DRN (Wilcox test for degree, *P*-value = 7.841e−08). We then randomly permuted the DRN and calculated the mean degree of COSMIC cancer genes for 1000 times. Based on the null distribution generated from randomly permuted DRN, the mean degree of COSMIC cancer genes in the original DRN is significantly larger (*P*-value = 0.002). These results illustrated that our quantitative method for regulatory intensity is feasible and effective to analyze gene dysregulation relevant to carcinogenesis.

### Compare the accuracy of 4-DysReg with other CRC signatures

We compared the predictive accuracy of 4-DysReg with previously reported CRC expression

signatures, including RUNX3 (Soong et al., 2009), ColoPrint (contains 18 genes) (Salazar et al., 2011), ColoGuideEx (contains 13 genes) (Agesen et al., 2012), ColoGuidePro (contains 7 genes) (Sveen et al., 2012), ColoFinder (contains 9 genes) (Shi and He, 2016), CRCassigner-30 (Sadanandam et al., 2013), CRCassigner-7 (Sadanandam et al., 2013), a 7-gene signature (Chen et al., 2017), and a 4-gene signature (Zou et al., 2015). It was noted that CRCassigner-30 and CRCassigner-7 had the association with prognosis and responses to therapy. ColoPrint contains 18 genes, including MCTP1, LAMA3, CTSC, PYROXD1, EDEM1, IL2RB, ZNF697, SLC6A11, IL2RA, CYFIP2, PIM3, LIF, PLIN3, HSD3B1, ZBED4, PPARA, THNSL2, CA4388O2 (SLC6A11 and CA4388O2 are not within the preprocessed TCGA CRC expression data). ColoFinder contains 9 genes, including APC, MLH1, MSH2, MSH6, TP53, TGFBR2, SMAD4, KRAS, PTEN. ColoGuideEx contains 13 genes, including PIGR, CXCL13, MMP3, TUBA1B, SESN1, AZGP1, KLK6, EPHA7, SEMA3A, DSC3, CXCL10, ENPP3, BNIP3. ColoGuidePro contains 7 genes, including OLFM4, CXCL9, DMBT1, UGT2B17, SEMA3A, NT5E, WNT11 (UGT2B17 is not within the preprocessed TCGA CRC expression data). CRCassigner-30 contains 30 genes, including LY6G6D, KRT23, CEL, ACSL6, EREG, CFTR, TCN1, PCSK1, NCRNA00261, SPINK4, REG4, MUC2, TFF3, CLCA4, ZG16, CA1, MS4A12, CA4, CXCL13, RARRES3, GZMA, IDO1, CXCL9, SFRP2, COL10A1, CYP1B1, MGP, MSRB3, ZEB1, FLNA (NCRNA00261 and CA1 are not within the preprocessed TCGA CRC expression data). CRCassigner-7 contians 7 genes, including SFRP2, ZEB1, FLNA, RARRES3, CFTR, MUC2, TFF3. A 7-gene signature contains NHLRC3, ZDHHC21, PRR14L, CCBL1, PTPRB, PNPO, PPIP5K2 (PRR14L is not within the preprocessed TCGA CRC expression data). A 4-gene signature contains CEACAM5, IL10, KPNA2, ENO2. All the genes among these signatures are within preprocessed GSE39582.

The comparison was implemented in TCGA CRC and GSE39582. In each dataset, 60% of the samples were randomly selected as training set to fit a cox model with each signature, and the left 40% were taken as testing set to calculate C-index. Cross-validation of each signature was repeated 100 times. The C-indexes of testing datasets were used to compare the accuracy of 4-DysReg and other seven CRC signatures.

**Validate the training set for predicting chemotherapeutic benefit with 4-DysReg**

Adjuvant chemotherapy (ADJC) is preferred for curing CRC patients, and the guideline of ADJC is established on pathologic stage (Watanabe et al., 2018). Stage III and IV CRC patients are routinely recommended to receive ADJC. Stage II CRC patients with high risk of recurrence also consider ADJC, but the usefulness of postoperative ADJC has not been proved (Watanabe et al., 2018).

Herein, we used a large-scale CRC dataset GSE39582 (Marisa et al., 2013) (553 cancer samples),

where the ADJC records span from stage II to stage IV, to explore the predictive power of our 4-DysReg signature for chemotherapeutic benefits. In GSE39582, 232 samples were recorded with ADJC, and 305 samples were recorded without ADJC. The first question is how to choose training set in GSE39582 for exploring the predictive power of 4-DysReg for ADJC. Three types of training set were tested: (1) using 90% samples without ADJC as trainging set, (2) using 90% samples without ADJC as training set, (3) using 50% samples with ADJC and 50% samples without ADJC as training set. Based on the seven genes incolved in 4-DysReg, an OS cox model on training set was fitted. The risk score for each sample in different types of testing set was calculated by using this model. Zero was used to cut the samples into positive risk score group and negative risk score group. Log-rank test was used two check the diference of OS between the two groups. The processes were repeated 100 times.

Based on the results in the following table, using samples without ADJC as trainging set shows most robust in training set and different types of testing set. Thus, we used samples without ADJC as training set to analyse the predictive power of 4-DysReg for chenotherpieuc benefit.

**The results of log-rank test in three types of training set.** The data indicate negative logarithm of *P*-value of log-rank test. Data are expressed as median (first quantile–third quantile) of 100 times of cross-validation.

| **Samples partitioning** | **－ log10 (log-rank test *P*-value)** |
|---|---|
| **Train model with 90% samples without ADJC** | |
| Training set | 2.61 (2.05–3.15) |
| All samples without ADJC | 2.78 (2.22–3.56) |
| All samples with ADJC | 2.43 (1.86–3.40) |
| All sampels in GSE39582 | 4.71 (3.81–5.42) |
| **Train model with 90% samples with ADJC** | |
| Training set | 3.16 (2.72–3.64) |
| All samples with ADJC | 3.26 (2.96–3.64) |
| All samples without ADJC | 1.75 (1.34–2.27) |
| All sampels in GSE39582 | 4.24 (3.51–4.75) |
| **Train model with 50% samples with ADJC and 50% samples without ADJC** | |
| Training set | 3.21 (2.58–4.23) |
| All samples excluded in training set | 1.20 (0.72–1.89) |
| All samples in GSE39582 | 3.81 (3.10–4.82) |

**Compare predictive power of 4-DysReg for chemotherapeutic benefit with other signatures**

We also compared the predictive power of 4-DysReg for chemotherapeutic benefit with other signatures, including ColoPrint (contains 18 genes) (Salazar et al., 2011), ColoGuideEx (contains 13 genes) (Agesen et al., 2012), ColoGuidePro (contains 7 genes) (Sveen et al., 2012), ColoFinder (contains 9 genes) (Shi and He, 2016), CRCassigner-30 (Sadanandam et al., 2013), CRCassigner-7 (Sadanandam et al., 2013), a 7-gene signature (Chen et al., 2017), and a 4-gene signature (Zou et al., 2015). According to the analysis of 4-DysReg in GSE39582 dataset, for each signature, an OS cox model was trained with the expression data of genes involved in the signature on samples without ADJC, which was used to calculate risk score of samples with ADJC. Zero was used to cut the samples into positive risk score group and negative risk score group. Log-rank test was used two check the diference of OS between the two groups. The results is listed in the following table. Excepting CRCassigner-7 for 5-FU, 4-DysReg performed much better for predicting chemotherapeutic benefit of all ADJC, combined ADJC, and 5-FU.

**The predictive power for chemotherapeutic benefit of different signatures in GSE39582.** all ADJC, the predictive power for all samples accepted ADJC; Combined ADJC, the predictive power for samples accepted combined chemotherapy (including FOLFIRI, FOLFOX, FUFOL); 5-FU, the predictive power for samples accepted 5-FU chemotherapy.

| Signature | all ADJC | Combined ADJC | 5-FU |
|---|---|---|---|
| 4-DysReg | 0.000334566 | 0.009611007 | 0.058820647 |
| ColoPrint | 0.357520376 | 0.775498206 | 0.691372056 |
| ColoFinder | 0.892391767 | 0.56052493 | 0.926634491 |
| ColoGuideEx | 0.057959072 | 0.354844623 | 0.291728967 |
| ColoGuidePro | 0.09233785 | 0.142860846 | 0.756460024 |
| CRCassigner-30 | 0.1081174705 | 0.067797156 | 0.34856405 |
| CRCassigner-7 | 0.0327007268 | 0.611242596 | 0.02882337 |
| gene.7 | 0.252428393 | 0.860392984 | 0.22401826 |
| gene.4 | 0.022925741 | 0.19434038 | 0.389242744 |

**Analyze the correlation between expression of genes involved in 4-DysReg and abundance of tissue-infiltrating immune cells**

The abundance of tissue-infiltrating cells were estimated by MCPcounter, which used cell-specific transcriptomic markers to quantify the absolute abundance of ten types of immune and stromal cell populations in tissues based on bulk transcriptomic data (Becht et al., 2016). The immune cell types include T cells, CD8 T cells, cytotoxic lymphocytes, NK cells, B lineage,

monocytic lineage, myeloid dendritic cells, neutrophils; the stromal cells include endothelial cells and fibroblasts. MCPcounter outputs an abundance score for each cell type in every sample.

It is appealing that several genes involved in 4-Dysreg possess immune-related functions, including RUNX3 (Ito et al., 2015), GPR15 (Habtezion et al., 2016; Pan et al., 2017), P2RY8 (Muppidi et al., 2014), SNAI3 (Dahlem et al., 2012), TLR7 (Schon and Schon, 2008; Zhang et al., 2016), and SIGLEC1 (Fraschilla and Pillai, 2017). We anaslyzed the correlation between the expression of these genes and the abundance of eight immune cell types, including T cells, CD8 T cells, cytotoxic lymphocytes, NK cells, B lineage, monocytic lineage, myeloid dendritic cells, neutrophils. The results showed that the expression of these genes exhibited positive correlation with the abundance of eight immune cell within CRC tissue (Fig. S5).

## References


Agesen, T.H., Sveen, A., Merok, M.A., et al. (2012). ColoGuideEx: a robust gene classifier specific for stage II colorectal cancer prognosis. Gut *61*, 1560-1567.

Becht, E., Giraldo, N.A., Lacroix, L., et al. (2016). Estimating the population abundance of tissue-infiltrating immune and stromal cell populations using gene expression. Genome Biol. *17*, 218.

Chen, H., Sun, X., Ge, W., et al. (2017). A seven-gene signature predicts overall survival of patients with colorectal cancer. Oncotarget *8*, 95054-95065.

Dahlem, T., Cho, S., Spangrude, G.J., et al. (2012). Overexpression of Snai3 suppresses lymphoid- and enhances myeloid-cell differentiation. Eur. J. Immunol *42*, 1038-1043.

Fraschilla, I., and Pillai, S. (2017). Viewing Siglecs through the lens of tumor immunology. Immunol. Rev. *276*, 178-191.

Habtezion, A., Nguyen, L.P., Hadeiba, H., et al. (2016). Leukocyte Trafficking to the Small Intestine and Colon. Gastroenterology *150*, 340-354.

Ito, Y., Bae, S.C., and Chuang, L.S. (2015). The RUNX family: developmental regulators in cancer. Nat. Rev. Cancer *15*, 81-95.

Marisa, L., de Reynies, A., Duval, A., et al. (2013). Gene expression classification of colon cancer into molecular subtypes: characterization, validation, and prognostic value. PLoS Med. *10*, e1001453.

Muppidi, J.R., Schmitz, R., Green, J.A., et al. (2014). Loss of signalling via Galpha13 in germinal centre B-cell-derived lymphoma. Nature *516*, 254-258.

Pan, B., Wang, X., Kojima, S., et al. (2017). The fifth epidermal growth factor like region of thrombomodulin alleviates LPS-induced sepsis through interacting with GPR15.

Thromb. Haemost. *117*, 570-579.

Sadanandam, A., Lyssiotis, C.A., Homicsko, K., et al. (2013). A colorectal cancer classification system that associates cellular phenotype and responses to therapy. Nat. Med. *19*, 619-625.

Salazar, R., Roepman, P., Capella, G., et al. (2011). Gene expression signature to improve prognosis prediction of stage II and III colorectal cancer. J. Clin. Oncol. *29*, 17-24.

Schon, M.P., and Schon, M. (2008). TLR7 and TLR8 as targets in cancer therapy. Oncogene *27*, 190-199.

Shi, M., and He, J. (2016). ColoFinder: a prognostic 9-gene signature improves prognosis for 871 stage II and III colorectal cancer patients. PeerJ. *4*, e1804.

Sondka, Z., Bamford, S., Cole, C.G., et al. (2018). The COSMIC Cancer Gene Census: describing genetic dysfunction across all human cancers. Nat. Rev. Cancer *18*, 696-705.

Soong, R., Shah, N., Peh, B.K., et al. (2009). The expression of RUNX3 in colorectal cancer is associated with disease stage and patient outcome. Br. J. Cancer. *100*, 676-679.

Sveen, A., Agesen, T.H., Nesbakken, A., et al. (2012). ColoGuidePro: a prognostic 7-gene expression signature for stage III colorectal cancer patients. Clin. Cancer Res. *18*, 6001-6010.

Watanabe, T., Muro, K., Ajioka, Y., et al. (2018). Japanese Society for Cancer of the Colon and Rectum (JSCCR) guidelines 2016 for the treatment of colorectal cancer. Int. J Clin. Oncol *23*, 1-34.

Zhang, Z., Ohto, U., Shibata, T., et al. (2016). Structural Analysis Reveals that Toll-like Receptor 7 Is a Dual Receptor for Guanosine and Single-Stranded RNA. Immunity *45*, 737-748.

Zou, M., Zhang, P.J., Wen, X.Y., et al. (2015). A novel mixed integer programming for multi-biomarker panel identification by distinguishing malignant from benign colorectal tumors. Methods *83*, 3-17.

## Supplementary Figures

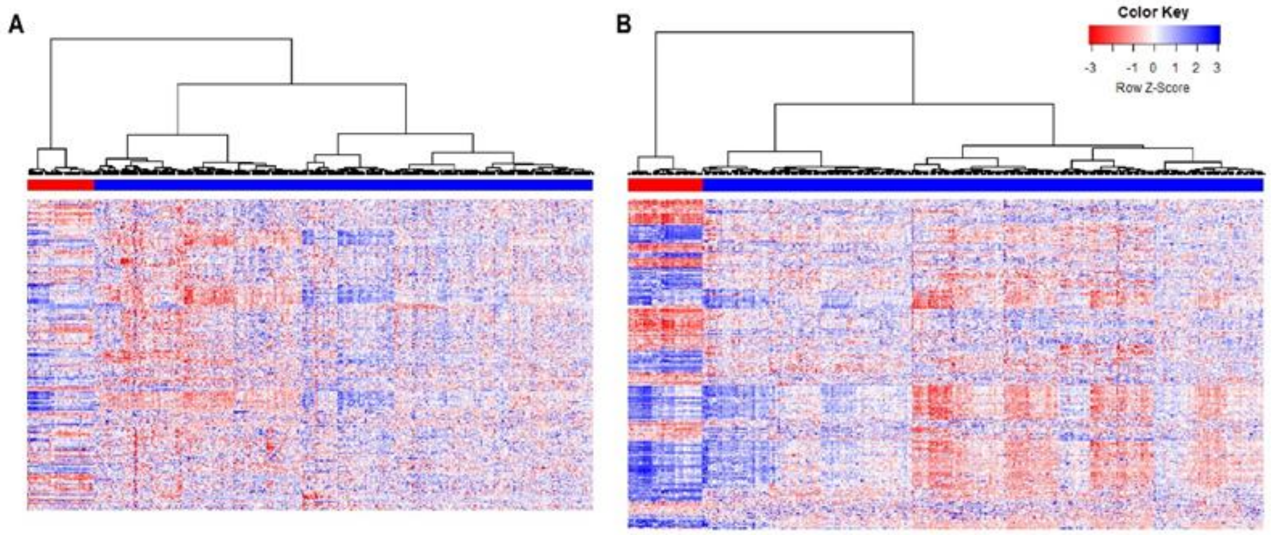


**Supplementary Figure S1** Clustering results of TFs (**A**) and targets (**B**) among the identified dysregulations in TCGA CRC dataset. Red bar represents normal samples, blue bar represents cancer samples. Both targets and TFs in dysregulations could correctly classify tumor and normal samples.

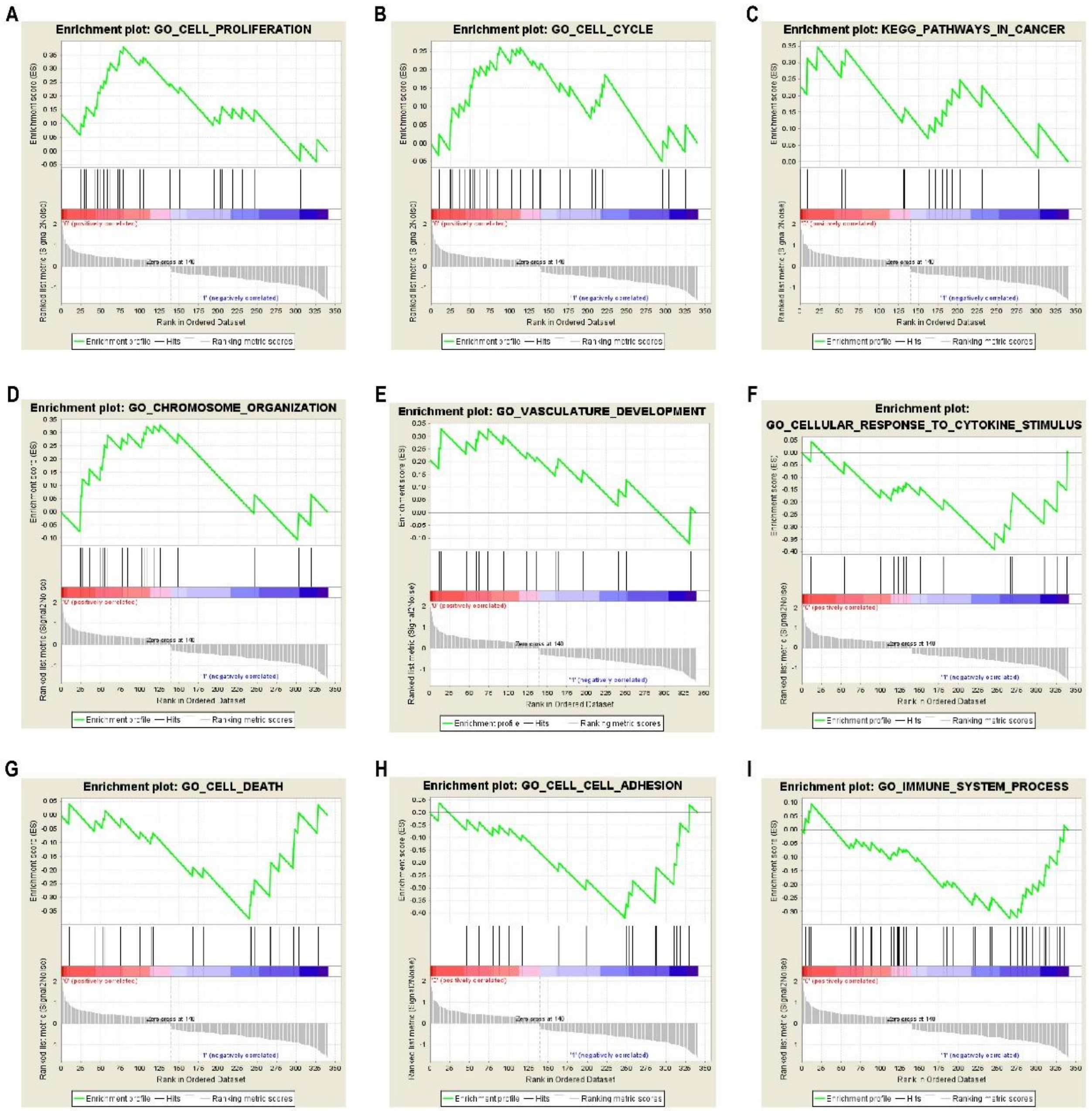


**Supplementary Figure S2** The results of GSEA with targets involved in the identified dysregulations. (**A**) Cell proliferation. (**B**) Cell cycle. (**C**) Pathways in cancer. (**D**) Chromosome organization. (**E**) Vasculature development. (**F**) Cellular response to cytokine stimulus. (**G**) Cell death. (**H**) Cell-cell adhesion. (**I**) Immune system process.

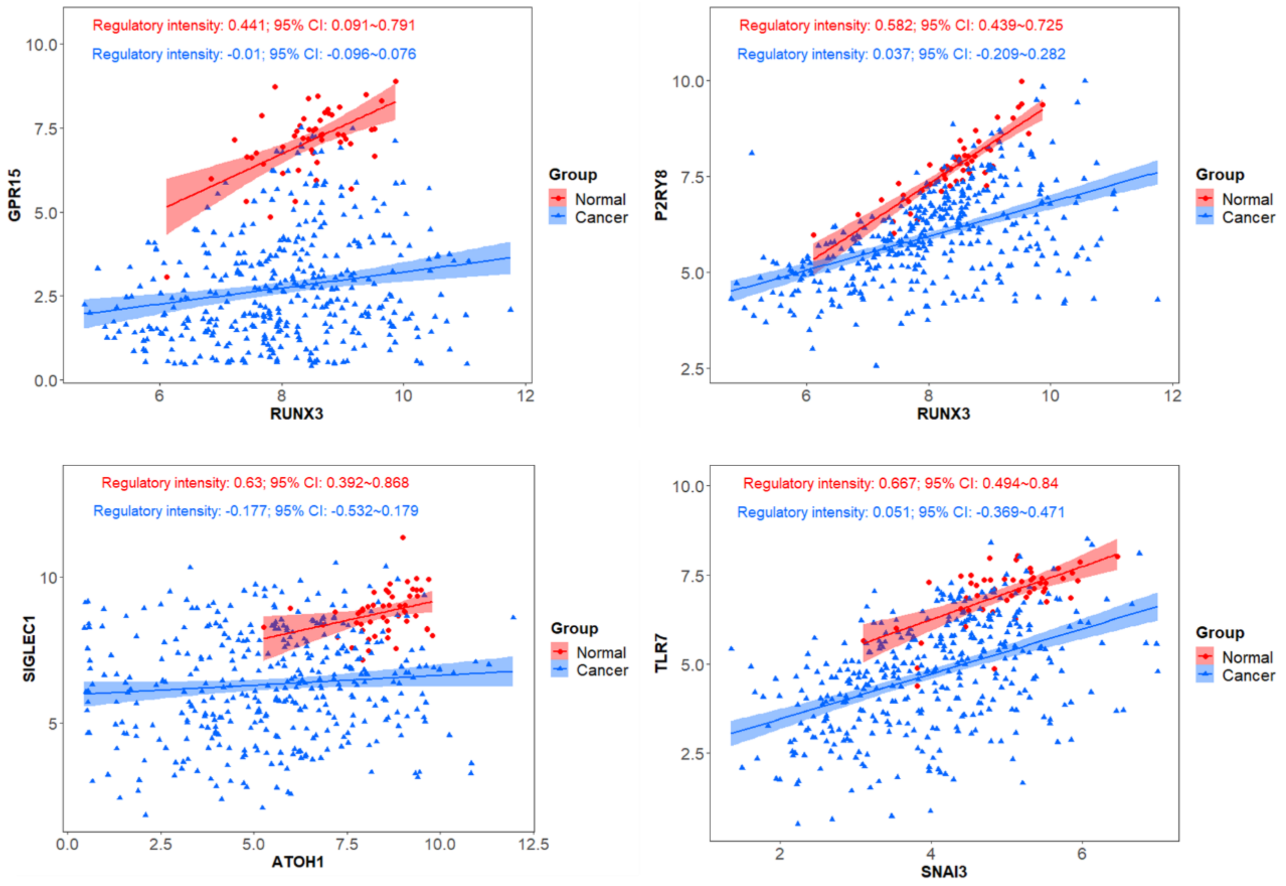


**Supplementary Figure S3** The expression pattern of gene dysregulations between normal and cancer. (**A**) RUNX3→GPR15. (**B**) RUNX3→P2RY8. (**C**) ATOH1→SIGLEC1. (**D**) SNAI3→TLR7. X-axis denotes TF's expression level and y-axis denotes target's expression level. One point corresponds to one sample, with red representing normal and blue representing cancer. The regression lines and confidence interval shadows were calculated by single variable regression and used to visualize the differences of gene regulation between conditions.

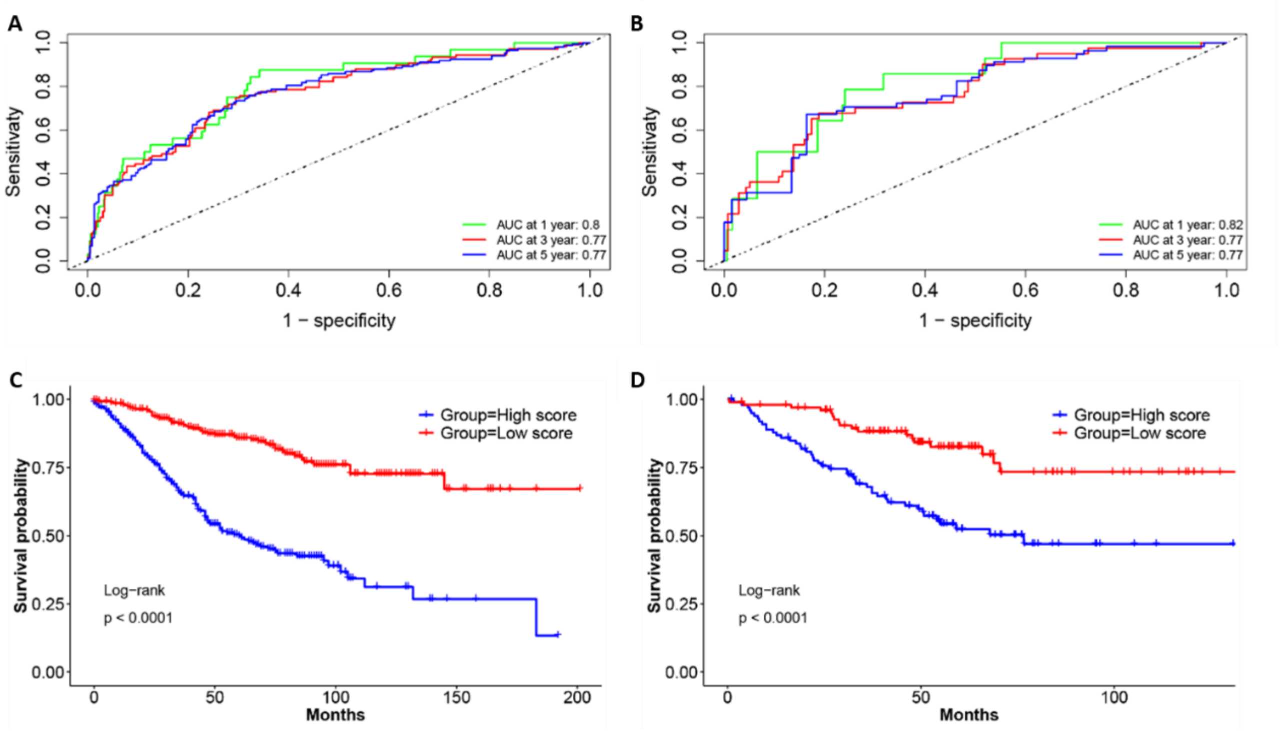


**Supplementary Figure S4** Testing the accuracy of 4-DysReg in GSE39582 and GSE17538. (**A-B**) Time-dependent ROC curves for risk score at 1-, 3-, 5- year survival in GSE39582 (**A**) and GSE17538 (**B**). (**C-D**) Kaplan–Meier curves of OS between two groups cut by median risk core in GSE39582 (**C**), and GSE17538 (**D**). *P*-value was generated from log-rank test. High score: the group with risk score larger than the median. Low score: the group with risk score smaller than the median.

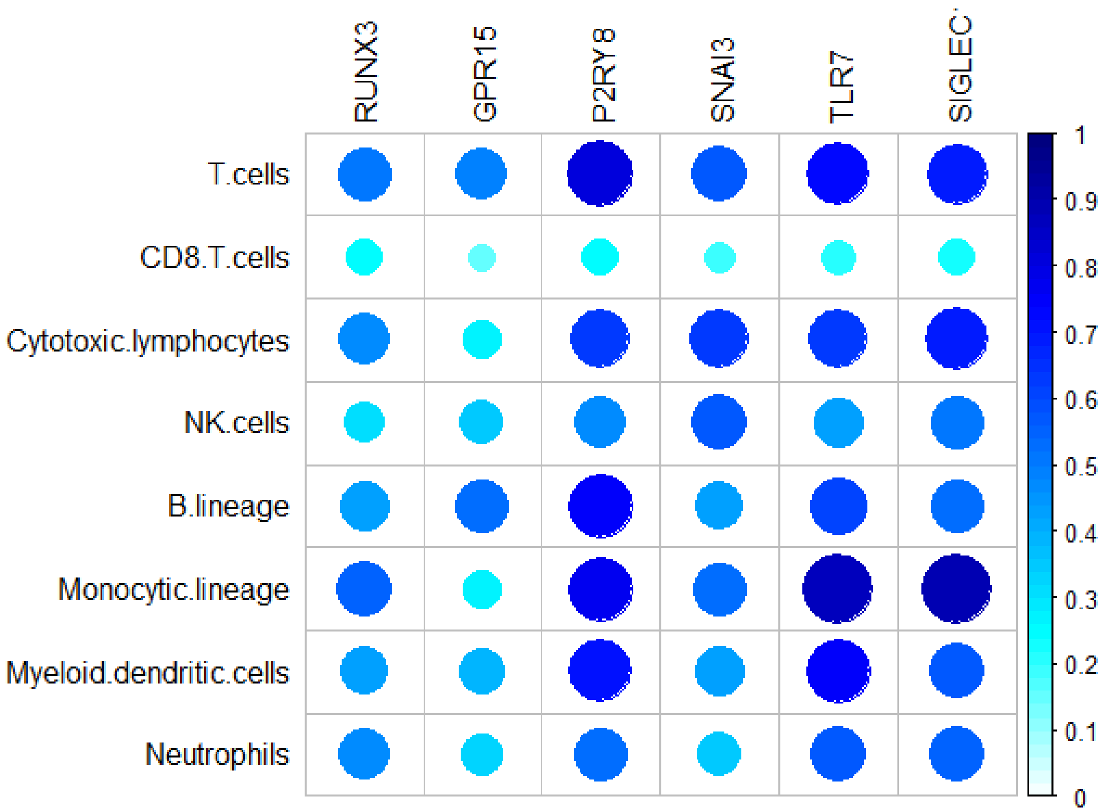


**Supplementary Figure S5** The correlation between expression of immune-related genes involved in 4-DysReg and abundance of eight immune cell types. The colors indicate spearman correlation coefficient.

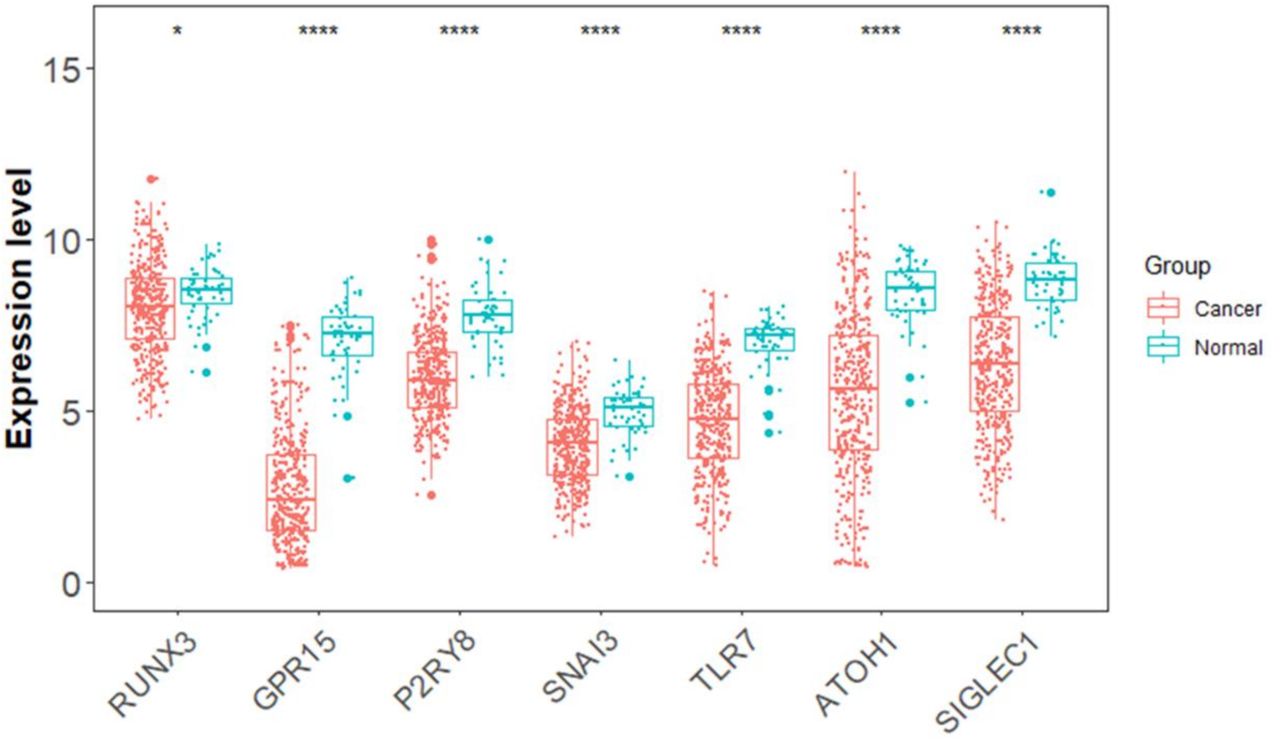


**Supplementary Figure S6** The expression of seven genes involved in 4-DysReg between normal and cancer in TCGA CRC dataset. The significance was generated from Wilcox test. **P*-value < 0.05, ** *P*-value < 0.01, *** *P*-value < 0.001.
